# Assessing the effects of alternative plant-based meats *v*. animal meats on biomarkers of inflammation: a secondary analysis of the SWAP-MEAT randomized crossover trial

**DOI:** 10.1017/jns.2022.84

**Published:** 2022-09-23

**Authors:** Anthony Crimarco, Matthew J. Landry, Matthew M. Carter, Christopher D. Gardner

**Affiliations:** 1 Stanford Prevention Research Center, Stanford School of Medicine, Stanford, CA, USA; 2 Microbiology and Immunology, Stanford School of Medicine, Stanford, CA, USA

**Keywords:** Dietary intervention, Inflammation, Nutrition, Plant-based meat, Red meat

## Abstract

Alternative plant-based meats have grown in popularity with consumers recently and researchers are examining the potential health effects, or risks, from consuming these products. Because there have been no studies to date that have specifically assessed the health effects of plant-based meats on biomarkers of inflammation, the purpose of this work was to conduct a secondary analysis of the Study With Appetizing Plantfood – Meat Eating Alternatives Trial (SWAP-MEAT). SWAP-MEAT was a randomised crossover trial that involved generally healthy adults eating 2 or more servings of plant-based meats per day for 8 weeks (i.e. Plant phase) followed by 2 or more servings of animal meats per day for 8 weeks (i.e. Animal phase). Results of linear mixed-effects models indicated only 4 out of 92 biomarkers reached statistical significance. The results were contrary to our hypothesis, since we expected relative improvements in biomarkers of inflammation from the plant-based meats.

## Introduction

Alternative plant-based proteins (or plant-based meats) are vegan or vegetarian products designed to resemble the taste and appearance of traditional burgers, sausages or other meats^(1)^. Based on a growing interest among consumers, several companies have created and marketed plant-based meat products to accelerate the shift away from traditional animal meats^(2)^. In the United States, the sales of plant-based meat products were over $900 million in 2019, and more than doubled in 2020^(3)^. The increased demand for plant-based meats is due to greater consumer interest in health, as well as environmental and animal welfare^(4)^. Additionally, some individuals that are interested in reducing their meat consumption find substituting plant-based meats to be a more manageable dietary modification compared with completely abstaining from meat products^(5,6)^.

Based on a paucity of data assessing the potential health benefits or risks of plant-based meats, we recently conducted a clinical trial to specifically compare the effects of consuming plant-based meats *v*. animal meats (mostly red meats) on emerging and traditional cardiometabolic risk factors among 36 generally healthy adults^(7)^.

The primary outcome of the Study With Appetizing Plantfoods – Meat Eating Alternatives Trial (SWAP-MEAT) study was serum concentrations of trimethylamine-N-oxide (TMAO), a molecule generated through gut metabolism that is associated with an increased risk for cardiovascular disease^(8)^. Recent trials have reported that red meat intake raises serum TMAO levels^(9–11)^. Observational research has indicated that vegans and vegetarians have lower TMAO levels compared with meat eaters^(11–13)^. In the SWAP-MEAT study, TMAO concentrations were significantly lower during the 8 weeks participants substituted the plant-based meats in place of the animal meats. Because TMAO is associated with inflammation^(14)^, we were interested in examining whether substituting plant-based meats over animal meats has a differential effect on other biomarkers of inflammation. While there is some evidence that consuming plant-based diets can improve inflammation^(15–17)^, there are no data specifically on the consumption of plant-based meats. Therefore, the purpose of the present study was to conduct a secondary analysis of the SWAP-MEAT study that assessed the effects of consuming plant-based meats *v*. animal meats on a broad range of biomarkers of inflammation^(18)^. We hypothesised that some of the biomarkers would be improved while consuming the plant-based meats.

## Methods

### Study design

The SWAP-MEAT (ClinicalTrial.gov registration number: NCT 03718988) was a randomised crossover design that involved participants eating at least 2 or more daily servings of plant-based meats for 8 weeks (Plant-based phase) followed by 2 or more daily servings of animal meats for 8 weeks (Animal phase) (or vice versa). A single serving size was 3–4 ounces per product. Complete details of the nutritional profile of the plant-based meat and animal meat products can be found in the published manuscript of the original study^(7)^. The animal meats used in the present study were supplied from a San Francisco-based organic foods delivery service; the red meat sources were grass-fed. The plant-based meats used in the present study were supplied from Beyond Meat. Both types of meats were provided to the participants throughout the study. Other than the study products, participants were instructed to keep all dietary habits as consistent as possible between the two phases, such that the only dietary changes were the consumption of the plant-based meat products or the animal-based meat products. Participants consented to allow any frozen and archived blood samples that were available after completing the main study analyses to be used to examine additional factors that were been specified in the original protocol. Complete details of the original study have been reported elsewhere^(7)^. Participant enrollment began on 5 December 2018 and continued through 9 July 2019. The date of final follow-up data collection was on 5 December 2019.

### Participants

To be eligible for this trial, participants were required to have a stable dietary history, defined as neither introducing nor eliminating a major food group in their diet for at least the previous month.

#### Randomisation

Randomisation to one of the two diet sequences (Plant→Animal *v*. Animal→Plant) was performed in block sizes of 4 by an independent statistician. Pairs (spousal or parent–child) were randomised in block sizes of 2. Participants did not learn of their diet sequence until they completed all baseline measures and surveys. Laboratory technicians and study staff conducting blood and stool analyses were blinded to the diet sequence.

Exclusion criteria were weighing <110 lbs (50 kg), having a body mass index (BMI) >40, LDL-C >190 mg/dl, systolic blood pressure (SBP) >160 mmHg or diastolic blood pressure (DBP) >90 mmHg, clinically significant or unstable pulmonary, cardiovascular, gastrointestinal, hepatic or renal functional abnormality, or pregnancy. Individuals were excluded or had their study start date delayed if within the past 2 months they took systemic antibiotics, antifungals, antivirals or antiparasitics, corticosteroids, cytokines, methotrexate or immunosuppressive cytotoxic agents known to affect the microbiome.

#### Participant details

The 36 participants who provided complete data for both crossover phases were general healthy adults; 67 % were women, 69 % were Caucasian, with an average of 50 ± 14 years and BMI of 28 ± 5 kg/m^2^ (Table 1).

**Table 1.** Study participants’ baseline characteristics

Variable	Category	Plant-based phase first: Animal phase second	Animal phase first: Plant-based phase second	All
Gender, No.	Female	15	9	24
	Male	3	9	12
Age, mean (sd), years		49·3 (11·7)	51·1 (16·0)	50·2 (13·8)
Highest level of education achieved, No.	Less than high school	0	1	1
	High school degree	1	0	1
	Some college	0	4	4
	College degree	7	7	14
	Some post-graduate school	2	1	3
	Post-graduate degree	8	5	13
Race/ethnicity, No.	Non-Hispanic White	11	14	25
	Hispanic/Latinx	3	0	3
	Asian	3	2	5
	Black/African American	1	0	1
	Others	0	2	2
Weight, mean (sd), kg	Women	73·7 (16·8)	71·5 (14·1)	72·8 (15·6)
	Men	80·9 (7·0)	93·2 (18·6)	88·7 (17·1)
	Both sexes	74·9 (15·7)	82·3 (19·5)	78·0 (17·6)
Body mass index, mean (sd), kg/m^2^	Women	27·7 (5·7)	27·0 (4·3)	27·4 (5·1)
	Men	26·2 (2·2)	29·7 (6·0)	28·8 (5·4)
	Both sexes	27·4 (5·2)	28·3 (5·3)	27·9 (5·2)

### Inflammatory biomarkers

Data for inflammatory biomarkers were collected from participants’ blood samples at baseline, week 8, and week 16. The data were analysed from Olink Proteomics using the 92 inflammation-related protein biomarker panel (https://www.olink.com/products/inflammation/). Specific inflammatory biomarkers were selected for primary analysis based on a review of previous literature regarding biomarkers that were both available within the Olink panel and that had been reported to change in response to increased consumption of plant-based foods: interleukin-6 (IL-6), interleukin-18 (IL-18) and tumour necrosis factor (TNF)^(15–17)^. We also selected three additional biomarkers that were reported to improve in previous nutrition studies in response to dietary change^(19)^. The other three biomarkers were: interleukin-12 subunit beta (IL-12B), interleukin-10 (IL-10) and transforming growth factor beta (TGF-β) for the main analysis based on these being some of the most commonly analysed biomarkers of inflammation from the literature on dietary patterns and inflammation (Table 2)^(19)^. The remaining eighty-six markers were analysed as a secondary analysis, and significance was assessed after controlling for a false discovery rate of <0·05^(20)^. Note that all biomarkers are expressed in units of normalised protein expression (NPX).

**Table 2.** Descriptive statistics of inflammatory biomarkers^a^

Inflammatory marker	Baseline: mean (sd)	Plant-based meat phase (week 8): mean (sd)	Animal meat phase (week 8): mean (sd)
Interleukin-6 (IL-6)	0·97 (0·60)	1·06 (0·56)	1·06 (0·68)
Interleukin-18 (IL-18)	7·85 (0·58)	7·90 (0·54)	7·92 (0·59)
Tumour necrosis factor (TNF)	1·28 (0·47)	1·32 (0·67)	1·34 (0·66)
Interleukin-12 subunit beta (IL-12B)	4·77 (0·53)	4·80 (0·55)	4·81 (0·51)
Interleukin-10 (IL-10)	2·18 (0·34)	2·23 (0·39)	2·25 (0·46)
Transforming growth factor beta (TGF-β)	4·92 (0·40)	4·96 (0·40)	4·92 (0·37)

a Biomarker units are normalised protein expression (NPX).

### Statistical analyses

Data were analysed in R version 3.6.3. Primary used for modelling were ‘lme4’ and ‘lmerTest’^(21–23)^. Linear mixed-effects models were conducted to compare changes in the biomarkers of inflammation from baseline and the end of each dietary phase (weeks 8 and 16) to determine if the changes were significantly different for the Plant-based phase compared to the Animal phase, adjusting for the fixed effect of diet order (i.e. study arm).

## Results

The participants ate similar amounts of calories, macronutrients and servings of plant-based meat or animal meat products (~2·5 and 2·6 servings per day, respectively) throughout the intervention. Complete details of adherence are available elsewhere^(7)^. Table 2 presents the mean values of the six biomarkers selected for the main analysis and Supplementary Table S1 presents the mean values for all of the biomarkers. Overall, none of the biomarkers in main analysis comparing the between-phase change scores reached statistical significance (Table 3 and Supplementary Table S2).

**Table 3.** Results of linear mixed-effects models comparing the change scores of selected inflammatory biomarkers from the two dietary phases^a^

Variables	Plant phase Mean ± se	Meat phase Mean ± se	Plant−Animal Mean Difference [95 % CI]		*P*-value^b^
IL-6	0·09 ± 0·11	0·10 ± 0·12	–0·01	–0·21 to 0·20	0·95
IL-18	0·05 ± 0·05	0·07 ± 0·05	–0·02	–0·12 to 0·08	0·66
TNF	0·05 ± 0·11	0·06 ± 0·11	–0·01	–0·12 to 0·09	0·80
IL-12B	0·03 ± 0·05	0·04 ± 0·05	–0·01	–0·12 to 0·11	0·93
IL-10	0·05 ± 0·07	0·07 ± 0·09	–0·02	–0·17 to 0·13	0·77
TGB-β	–0·09 ± 0·05	–0·02 ± 0·05	–0·07	–0·16 to 0·02	0·14

a Biomarker units are normalised protein expression (NPX).

b *P*-value for the Likelihood ratio test from mixed-effects model evaluating change scores for each product type (Plant compared with Animal), adjusting for order and phase.

For the paired within-phase scores, only four biomarkers reached statistical significance. Interleukin-7 (IL7) (+0·15 ± 0·07, *P* = 0·01), Neurotrophin-3 (NT-3) (+0·17 ± 0·07, *P* =0·01), changed from baseline to the end of the Plant-based phase (Supplementary Table S3). FMS-like tyrosine kinase 3 ligand (FLT3L) (+0·12 ± 0·05, *P* = 0·02) (Supplementary Table S4). Interleukin-22 receptor, alpha 1 (IL22RA1) increased in both phases (+0·28 ± 0·11, *P* = 0·01 in the plant-based meat phase) and (+0·21 ± 0·09, *P* = 0·03 in the animal meat phase).

## Discussion and conclusion

This secondary analysis of the SWAP-MEAT randomised crossover trial assessed changes in biomarkers of inflammation when participants ate two or more daily servings of plant-based meats *v*. animal meats for 8 weeks each. Overall, none of the change scores between the two diet phases were significantly different, and for the within-phase paired scores, only four out of ninety-two inflammatory biomarkers reached statistical significance. Additionally, the four biomarkers all slightly increased regardless of diet phase and order. These results do not support our hypothesis, that biomarkers of inflammation would improve during the plant-based meat diet. It could be that the duration of the diet protocols was not long enough to observe any notable changes. In Menzel *et al.*'s review of plant-based diets and their association with biomarkers of inflammation, the authors noted that there were no significant changes in C-reactive protein (CRP) concentrations for any of the studies that assessed vegetarian diets for a duration of 6 months or less^(17)^. Their sensitivity analysis indicated that the impact of vegetarian diets on CRP was more obvious for participants following the diet for at least 10 years^(17)^. While CRP is not included in the Olink panel, some of the other biomarkers in our analysis (IL-6 and TNF) were also reported to improve in previous meta-analyses^(15–17)^; therefore, it is possible that the length of time for substituting plant-based meats in place of red meats within this study was too short to achieve detectable improvements in systemic inflammation^(24)^.

A second explanation for the modest changes in biomarkers of inflammation is that the mere substitution of plant-based meats over animal meats is not sufficient enough by itself to improve systematic inflammation. Only replacing animal meats (which comprised ~25 % of our participants’ energy intake in this study), but keeping all other dietary components the same, may not be enough to improve chronic inflammation, since the majority of participants’ foods were self-selected and likely did not change during the intervention. An overall healthy, plant-based diet may have yielded different results, since the focus of plant-based diets is to maximise the consumption of nutrient-dense foods like fruits, vegetables, nuts, wholegrains and seeds, while minimising the consumption of processed foods and animal foods^(25)^. In general, highly processed foods are associated with increased weight gain and an increased risk for a number of leading chronic health conditions. The plant-based meat products used in our study would fit the definition of ‘ultra-processed’ foods, due to a number of added ingredients used to enhanced the flavor and texture of the meats. Therefore, it is possible that the plant-based meats may not have any benefit to improving inflammatory biomarkers due to the products being highly processed. However, others have noted that plant-based meats ‘are often perceived as low-quality, ultra-processed foods. However, we argue that the mere industrial processing of ingredients of plant origin does not make a PBMA product ultra-processed by default^(26)^.’ Additionally, recent work conducted by van Valiet *et al.* on the metabolomic profiling that compared a plant-based beef substitute to grass-fed beef demonstrates the complexity in trying to compare and understand nutritional adequacy of plant-based meat products to animal meats, since both foods assessed both contained and lacked differing nutrients with potential benefits to health^(27)^.

Other potential benefits of substituting plant-based for animal meats have been reported. A recent randomised clinical trial noted positive changes in the gut microbiome among participants that substituted ~4–6 servings of plant-based meats per week in place of animal meats^(26)^. Also, reductions in animal meat consumption, particularly red and processed meats, have been widely reported to have beneficial impacts on environmental sustainability^(28)^.

The strengths of the SWAP-MEAT study were minimal missing data from all participants, the crossover design of this trial allowed each participant to serve as their own control, and the strong dietary adherence to both dietary conditions. There were also some limitations to the study. Only the plant-based meat and animal meat products were controlled, while the remainder of the diet was self-selected, which limits the researchers’ control over the portion sizes or specific food selection. However, this does allow for increased external validity and generalizability. Our sample was ~66 % female and existing research on gender differences in inflammation and immune functioning suggest that women have higher levels of inflammation and poorer immune functioning^(29,30)^. This may impact the generalizability and interpretation of our results. Finally, the present study utilised only Beyond Meat products. There are numerous plant-based meat products with different ingredients or formulations, therefore the findings may not be generalisable to the consumption of other plant-based meat products.

In conclusion, while the results of the main trial indicated several improvements in CVD risk factors, including TMAO, for the plant-based meats, no differences in the selected biomarkers of inflammation were observed^(7)^. Future research may benefit from longer study duration periods.

## Supplementary material

For supplementary material accompanying this paper visit https://doi.org/10.1017/jns.2022.84. click here to view supplementary material
