# Supplementary material for: Assessing the effects of alternative plant-based meats v. animal meats on biomarkers of inflammation: a secondary analysis of the SWAP-MEAT randomized crossover trial
Source: J Nutr Sci. 2022 Sep 23;11:e82. doi: 10.1017/jns.2022.84 (PMC9554424; doi:10.1017/jns.2022.84)
Supplement: Supplementary file 1 [file S2048679022000842sup001.docx]

**Supplementary Table 1.** Descriptive statistics (mean, (SD)) for all inflammatory biomarkers.^a^

| **Variables** | **Baseline** | **Plant-based phase week 8** | **Animal phase week 8** |
| --- | --- | --- | --- |
| n | 35 | 35 | 35 |
| Adenosine Deaminase (ADA) (mean (SD)) | 4.21 (0.42) | 4.29 (0.39) | 4.27 (0.45) |
| Aretmin (ARTN) (mean (SD)) | -0.20 (0.38) | -0.23 (0.42) | -0.19 (0.37) |
| Axin-1 (AXIN1) (mean (SD)) | 0.37 (0.65) | 0.55 (0.80) | 0.37 (0.52) |
| Beta-nerve growth factor (Beta-NGF) (mean (SD)) | -0.50 (0.11) | -0.49 (0.12) | -0.53 (0.11) |
| Caspase-8 (CASP-8) (mean (SD)) | 0.49 (0.40) | 0.53 (0.41) | 0.54 (0.35) |
| Eotaxin (CCL11) (mean (SD)) | 7.03 (0.45) | 7.06 (0.44) | 7.05 (0.44) |
| C-C motif chemokine 19 (CCL19) (mean (SD)) | 8.41 (0.74) | 8.42 (0.78) | 8.26 (0.63) |
| C-C motif chemokine 20 (CCL20) (mean (SD)) | 5.73 (0.67) | 5.79 (0.72) | 5.88 (0.87) |
| C-C motif chemokine 23 (CCL23) (mean (SD)) | 9.57 (0.45) | 9.58 (0.51) | 9.62 (0.45) |
| C-C motif chemokine 25 (CCL25) (mean (SD)) | 4.99 (0.57) | 5.09 (0.58) | 5.03 (0.62) |
| C-C motif chemokine 28 (CCL28) (mean (SD)) | 0.92 (0.35) | 0.98 (0.35) | 0.93 (0.30) |
| C-C motif chemokine 3 (CCL3)(mean (SD)) | 5.19 (0.61) | 5.16 (0.41) | 5.18 (0.50) |
| C-C motif chemokine 4 (CCL4) (mean (SD)) | 6.04 (0.67) | 5.99 (0.52) | 6.00 (0.61) |
| Natural killer cell receptor 2B4 (CD244) (mean (SD)) | 4.69 (0.34) | 4.75 (0.26) | 4.71 (0.31) |
| CD40L receptor (CD40) (mean (SD)) | 9.86 (0.35) | 9.90 (0.33) | 9.89 (0.30) |
| T-cell surface glycoprotein CD5 (CD5) (mean (SD)) | 4.10 (0.31) | 4.12 (0.30) | 4.12 (0.31) |
| T-cell surface glycoprotein CD6 isoform (CD6) (mean (SD)) | 4.65 (0.44) | 4.72 (0.45) | 4.68 (0.51) |
| T cell surface glycoprotein CD8 alpha chain (CD8A) (mean (SD)) | 7.96 (0.63) | 7.97 (0.62) | 7.95 (0.62) |
| CUB domain-containing protein 1 (CDCP1) (mean (SD)) | 1.03 (0.54) | 1.10 (0.50) | 1.07 (0.53) |
| Cystatin D (CST5) (mean (SD)) | 5.34 (0.52) | 5.40 (0.49) | 5.40 (0.53) |
| Fractalkine (CX3CL1) (mean (SD)) | 2.77 (0.31) | 2.81 (0.30) | 2.84 (0.31) |
| C-X-C motif chemokine 1 (CXCL1) (mean (SD)) | 8.78 (0.84) | 8.81 (0.76) | 8.70 (0.74) |
| C-X-C motif chemokine 10 (CXCL10) (mean (SD)) | 9.04 (0.68) | 8.97 (0.46) | 9.08 (0.69) |
| C-X-C motif chemokine 11 (CXCL11) (mean (SD)) | 6.72 (0.82) | 6.57 (0.72) | 6.61 (0.81) |
| C-X-C motif chemokine 5 (CXCL5) (mean (SD)) | 11.51 (0.82) | 11.48 (0.70) | 11.57 (0.70) |
| C-X-C motif chemokine 6 (CXCL6) (mean (SD)) | 7.95 (0.82) | 7.88 (0.71) | 7.92 (0.76) |
| C-X-C motif chemokine 9 (CXCL9) (mean (SD)) | 5.59 (0.65) | 5.58 (0.61) | 5.63 (0.87) |
| Delta and Notch-like epidermal growth factor-related receptor (DNER) (mean (SD)) | 7.57 (0.26) | 7.61 (0.26) | 7.61 (0.31) |
| Protein S100-A12 (EN-RAGE) (mean (SD)) | 3.62 (1.01) | 3.74 (0.88) | 3.75 (0.81) |
| Fibroblast growth factor 19 (FGF-19) (mean (SD)) | 7.32 (1.06) | 7.23 (0.76) | 7.39 (0.92) |
| Fibroblast growth factor 21 (FGF-21) (mean (SD)) | 3.87 (1.35) | 3.84 (1.56) | 3.81 (1.61) |
| Fibroblast growth factor 23 (FGF-23) (mean (SD)) | -0.11 (0.27) | -0.12 (0.27) | -0.15 (0.31) |
| Fibroblast growth factor 5 (FGF-5) (mean (SD)) | -0.10 (0.29) | -0.04 (0.30) | -0.04 (0.29) |
| Fms-related tyrosine-kinase 3 ligand (FLT3L) (mean (SD)) | 7.78 (0.44) | 7.86 (0.50) | 7.90 (0.46) |
| Eukaryotic translation initiation factor 4E-binding protein 1 (4E-BP1) (mean (SD)) | 6.43 (0.92) | 6.72 (1.24) | 6.71 (1.05) |
| Glial cell line-dervied neurotrophic factor (GDNF) (mean (SD)) | 1.02 (0.58) | 0.91 (0.27) | 0.92 (0.31) |
| Hepatocyte growth factor (HGF) (mean (SD)) | 7.67 (0.47) | 7.76 (0.47) | 7.66 (0.43) |
| Interferon gamma (IFN-gamma) (mean (SD)) | 6.46 (1.38) | 6.24 (0.77) | 6.37 (1.12) |
| Interleukin-1 alpha (IL-1 alpha) (mean (SD)) | -0.63 (0.33) | -0.56 (0.44) | -0.47 (0.98) |
| Interleukin-10 (IL-10) (mean (SD)) | 2.18 (0.34) | 2.23 (0.39) | 2.25 (0.46) |
| Interleukin-10 receptor subunit alpha (IL-10RA) (mean (SD)) | -0.21 (0.83) | -0.27 (0.74) | -0.22 (0.74) |
| Interleukin-10 receptor subunit beta (IL-10RB) (mean (SD)) | 5.10 (0.30) | 5.15 (0.33) | 5.14 (0.31) |
| Interleukin-12 subunit beta (IL-12B) (mean (SD)) | 4.77 (0.53) | 4.80 (0.55) | 4.81 (0.51) |
| Interleukin-13 (IL-13) (mean (SD)) | -0.20 (0.64) | -0.22 (0.68) | -0.21 (0.63) |
| Interleukin-15 subunit alpha (IL-15RA) (mean (SD)) | -0.24 (0.32) | -0.18 (0.27) | -0.17 (0.25) |
| Interleukin-17A (IL-17A) (mean (SD)) | 0.26 (0.78) | 0.33 (0.76) | 0.28 (0.81) |
| Interleukin-17C (IL-17C) (mean (SD)) | 1.90 (0.77) | 2.02 (0.85) | 1.99 (0.88) |
| Interleukin-18 (IL-18) (mean (SD)) | 7.85 (0.58) | 7.90 (0.54) | 7.92 (0.59) |
| Interleukin-18 receptor 1 (IL-18R1) (mean (SD)) | 7.22 (0.36) | 7.20 (0.42) | 7.21 (0.42) |
| Interleukin-2 (IL-2) (mean (SD)) | -0.48 (0.25) | -0.42 (0.32) | -0.48 (0.29) |
| Interleukin-20 (IL-20) (mean (SD)) | -0.94 (0.32) | -0.91 (0.33) | -0.95 (0.28) |
| Interleukin-20 subunit alpha (IL-20RA) (mean (SD)) | -0.47 (0.35) | -0.48 (0.33) | -0.40 (0.31) |
| Interleukin-22 subunit alpha-1 (IL-22RA1) (mean (SD)) | 0.47 (0.59) | 0.75 (0.56) | 0.69 (0.62) |
| Interleukin-24 (IL-24) (mean (SD)) | -0.19 (0.90) | -0.21 (0.91) | -0.36 (0.97) |
| Interleukin-2 receptor subunit beta (IL-2RB) (mean (SD)) | -0.16 (0.38) | -0.20 (0.40) | -0.18 (0.39) |
| Interleukin-33 (IL-33) (mean (SD)) | -0.92 (0.35) | -0.96 (0.32) | -0.87 (0.28) |
| Interleukin-4 (IL-4) (mean (SD)) | -1.18 (0.49) | -1.20 (0.50) | -1.21 (0.41) |
| Interleukin-5 (IL-5) (mean (SD)) | -0.54 (0.74) | -0.66 (0.61) | -0.47 (0.82) |
| Interleukin-6 (IL-6) (mean (SD)) | 0.97 (0.60) | 1.06 (0.56) | 1.06 (0.68) |
| Interleukin-7 (IL-7) (mean (SD)) | 2.48 (0.47) | 2.63 (0.45) | 2.56 (0.38) |
| Interleukin-8 (IL-8) (mean (SD)) | 5.22 (0.59) | 5.32 (0.54) | 5.31 (0.54) |
| Latency-associated peptide transforming growth factor beta-1 (LAPTGF-beta-1) (mean (SD)) | 4.92 (0.40) | 4.96 (0.40) | 4.92 (0.37) |
| Leukemia inhibitory factor receptor (LIF-R) (mean (SD)) | -1.34 (0.43) | -1.31 (0.39) | -1.35 (0.35) |
| Leukemia inhibitory factor (LIF) (mean (SD)) | 2.78 (0.21) | 2.83 (0.24) | 2.84 (0.20) |
| Macrophage colony-stimulating factor 1 (CSF-1) (mean (SD)) | 11.16 (0.62) | 11.14 (0.52) | 11.19 (0.53) |
| Monocyte chemotactic protein 2 (MCP-2) (mean (SD)) | 8.47 (0.83) | 8.43 (0.75) | 8.46 (0.69) |
| Monocyte chemotactic protein 3 (MCP-3) (mean (SD)) | 0.62 (0.55) | 0.71 (0.54) | 0.78 (0.51) |
| Monocyte chemotactic protein 4 (MCP-4) (mean (SD)) | 14.17 (0.81) | 14.17 (0.78) | 14.15 (0.73) |
| Matrix metalloproteinase-1 (MMP-1) (mean (SD)) | 13.91 (0.84) | 13.94 (0.90) | 13.84 (0.82) |
| Matrix metalloproteinase-10 (MMP-10) (mean (SD)) | 8.49 (0.78) | 8.45 (0.69) | 8.41 (0.75) |
| Neuturin (NRTN) (mean (SD)) | -0.72 (0.57) | -0.71 (0.58) | -0.59 (0.50) |
| Neurotrophin-3 (NT-3) (mean (SD)) | 0.30 (0.43) | 0.47 (0.48) | 0.42 (0.45) |
| Osteoprotegerin (OPG) (mean (SD)) | 9.37 (0.37) | 9.45 (0.40) | 9.42 (0.44) |
| Oncostatin-M (OSM) (mean (SD)) | 4.23 (1.03) | 4.44 (1.00) | 4.26 (0.97) |
| Programmed cell death 1 ligand 1 (PD-L1) (mean (SD)) | 4.24 (0.40) | 4.26 (0.36) | 4.26 (0.39) |
| Stem cell factor (SCF) (mean (SD)) | 8.58 (0.35) | 8.64 (0.32) | 8.54 (0.35) |
| SIR-like protein 2 (SIRT2) (mean (SD)) | 1.20 (0.68) | 1.32 (1.12) | 1.36 (0.82) |
| Signaling lympohcytic activation molecule (SLAMF1) (mean (SD)) | 1.08 (0.37) | 1.12 (0.37) | 1.02 (0.41) |
| Sulfotransferase 1A1 (ST1A1) (mean (SD)) | 0.37 (0.70) | 0.49 (0.88) | 0.39 (0.84) |
| STAM-binding protein (STAMBP) (mean (SD)) | 2.23 (0.48) | 2.46 (0.79) | 2.31 (0.55) |
| Transforming growth factor alpha (TGF-alpha) (mean (SD)) | 2.30 (0.78) | 2.40 (0.81) | 2.30 (0.74) |
| Tumor necrosis factor (TNF) (mean (SD)) | 1.28 (0.47) | 1.32 (0.67) | 1.34 (0.66) |
| TNF-beta (TNF-β) (mean (SD)) | 3.65 (0.34) | 3.56 (0.33) | 3.63 (0.40) |
| Tumor necrosis factor receptor superfamily member 9 (mean (SD)) | 5.65 (0.48) | 5.70 (0.44) | 5.65 (0.49) |
| Tumor necrosis factor ligand superfamily member 14 (TNFSF14) (mean (SD)) | 3.56 (0.84) | 3.62 (0.84) | 3.46 (0.78) |
| TNF-related apoptosis-inducing ligand (TRAIL) (mean (SD)) | 7.08 (0.25) | 7.04 (0.28) | 7.07 (0.32) |
| TNF-related activation-induced cytokine (TRANCE) (mean (SD)) | 3.64 (0.64) | 3.50 (0.72) | 3.66 (0.70) |
| Thymic stromal lymphopoietin (TSLP) (mean (SD)) | 0.18 (0.52) | 0.17 (0.70) | 0.01 (0.80) |
| Tumor necrosis factor Ligand superfamily, member12 (TWEAK) (mean (SD)) | 8.23 (0.29) | 8.23 (0.34) | 8.25 (0.35) |
| Urokinase-type plasminogen activator (uPA) (mean (SD)) | 8.94 (0.29) | 8.97 (0.27) | 8.97 (0.34) |
| Vascular endothelial growth factor A (VEGF-A) (mean (SD)) | 10.18 (0.69) | 10.27 (0.70) | 10.20 (0.68) |

^a^Biomarker units are normalized protein expression (NPX)

**Supplementary Table 2.** Results of liner mixed effects models comparing the change scores of all inflammatory biomarkers.^a^

| **Variables** | **Plant-based phase ± SE** | **Animal phase Mean ± SE** | **Plant-Animal Mean Difference [95% CI]** | | **P-Value^b^** |
| --- | --- | --- | --- | --- | --- |
| Adenosine Deaminase (ADA) | 0.08 ± 0.05 | 0.06 ± 0.05 | 0.02 | -0.09 to 0.13 | 0.69 |
| Aretmin (ARTN) | -0.03 ± 0.07 | 0.01 ± 0.06 | -0.05 | -0.15 to 0.06 | 0.39 |
| Axin-1 (AXIN1) | 0.18 ± 0.15 | 0.0 ± 0.13 | 0.18 | -0.15 to 0.51 | 0.28 |
| Beta-nerve growth factor (Beta-NGF) | 0.10 ± 0.03 | -0.03 ± 0.03 | 0.04 | -0.01 to 0.09 | 0.07 |
| Caspase-8 (CASP-8) | 0.05 ± 0.07 | 0.06 ± 0.05 | -0.01 | -0.16 to 0.15 | 0.94 |
| Eotaxin (CCL11) | 0.03 ± 0.07 | 0.02 ± 0.06 | 0.01 | -0.16 to 0.18 | 0.91 |
| C-C motif chemokine 19 (CCL19) | 0.01 ± 0.10 | -0.14 ± 0.12 | -0.15 | -0.11 to 0.41 | 0.24 |
| C-C motif chemokine 20 (CCL20) | 0.05 ± 0.13 | 0.15 ± 0.15 | -0.10 | -0.40 to 0.21 | 0.53 |
| C-C motif chemokine 23 (CCL23) | 0.01 ± 0.07 | 0.05 ± 0.07 | -0.04 | -0.22 to 0.13 | 0.33 |
| C-C motif chemokine 25 (CCL25) | 0.09 ± 0.05 | 0.03 ± 0.04 | 0.06 | -0.07 to 0.19 | 0.06 |
| C-C motif chemokine 28 (CCL28) | 0.06 ± 0.06 | 0.01 ± 0.07 | 0.05 | -0.05 to 0.15 | 0.30 |
| C-C motif chemokine 3 (CCL3)(mean (SD)) | -0.03 ± 0.07 | -0.01 ± 0.09 | -0.02 | -0.13 to 0.17 | 0.80 |
| C-C motif chemokine 4 (CCL4) | -0.05 ± 0.08 | -0.04 ± 0.10 | -0.01 | -0.19 to 0.17 | 0.92 |
| Natural killer cell receptor 2B4 (CD244) | 0.06 ± 0.04 | 0.02 ± 0.04 | 0.03 | -0.05 to 0.12 | 0.43 |
| CD40L receptor (CD40) | 0.04 ± 0.05 | 0.03 ± 0.05 | 0.01 | -0.12 to 0.13 | 0.92 |
| T-cell surface glycoprotein CD5 (CD5) | 0.02 ± 0.04 | 0.03 ± 0.04 | -0.01 | -0.10 to 0.08 | 0.87 |
| T-cell surface glycoprotein CD6 isoform (CD6) | 0.07 ± 0.05 | 0.03 ± 0.05 | 0.04 | -0.08 to 0.15 | 0.53 |
| T cell surface glycoprotein CD8 alpha chain (CD8A) | 0.02 ± 0.05 | -0.01 ± 0.05 | 0.02 | -0.10 to 0.15 | 0.71 |
| CUB domain-containing protein 1 (CDCP1) | 0.08 ± 0.05 | 0.05 ± 0.05 | 0.03 | -0.7 to 0.13 | 0.51 |
| Cystatin D (CST5) | 0.06 ± 0.04 | 0.05 ± 0.05 | 0.01 | -0.11 to 0.12 | 0.92 |
| Fractalkine (CX3CL1) | 0.04 ± 0.05 | 0.07 ± 0.05 | -0.03 | -0.12 to 0.06 | 0.55 |
| C-X-C motif chemokine 1 (CXCL1) | 0.03 ± 0.10 | -0.08 ± 0.11 | 0.11 | -0.13 ± 0.34 | 0.35 |
| C-X-C motif chemokine 10 (CXCL10) | -0.07 ± 0.10 | 0.04 ± 0.15 | -0.11 | -0.34 ± 0.13 | 0.37 |
| C-X-C motif chemokine 11 (CXCL11) | -0.15 ± 0.12 | -0.11 ± 0.15 | -0.04 | -0.29 to 0.21 | 0.75 |
| C-X-C motif chemokine 5 (CXCL5) | -0.03 ± 0.07 | 0.07 ± 0.09 | -0.10 | -0.28 to 0.09 | 0.31 |
| C-X-C motif chemokine 6 (CXCL6) | -0.07 ± 0.07 | -0.04 ± 0.10 | -0.03 | -0.24 to 0.18 | 0.76 |
| C-X-C motif chemokine 9 (CXCL9) | -0.01 ± 0.09 | 0.04 ± 0.14 | -0.05 | -0.30 to 0.19 | 0.66 |
| Delta and Notch-like epidermal growth factor-related receptor (DNER) | 0.04 ± 0.04 | 0.04 ± 0.04 | 0.00 | -0.08 to 0.09 | 0.97 |
| Protein S100-A12 (EN-RAGE) | 0.12 ± 0.15 | 0.13 ± 0.15 | -0.01 | -0.33 to 0.32 | 0.96 |
| Fibroblast growth factor 19 (FGF-19) | -0.09 ± 0.14 | 0.08 ± 0.18 | -0.17 | -0.45 to 0.11 | 0.23 |
| Fibroblast growth factor 21 (FGF-21) | -0.03 ± 0.22 | -0.06 ± 0.25 | 0.03 | -0.30 to 0.35 | 0.84 |
| Fibroblast growth factor 23 (FGF-23) | -0.01 ± 0.01 | -0.02 ± 0.01 | 0.03 | -0.08 to 0.14 | 0.55 |
| Fibroblast growth factor 5 (FGF-5) | 0.06 ± 0.04 | 0.05 ± 0.04 | 0.01 | -0.08 to 0.10 | 0.86 |
| Fms-related tyrosine-kinase 3 ligand (FLT3L) | 0.09 ± 0.04 | 0.12 ± 0.05 | -0.03 | -0.13 to 0.07 | 0.51 |
| Eukaryotic translation initiation factor 4E-binding protein 1 (4E-BP1) | 0.29 ± 0.22 | 0.28 ± 0.24 | 0.01 | -0.54 to 0.51 | 0.98 |
| Glial cell line-dervied neurotrophic factor (GDNF) | -0.11 ± 0.10 | -0.10 ± 0.10 | -0.02 | -0.12 to 0.09 | 0.72 |
| Hepatocyte growth factor (HGF) | 0.09 ± 0.06 | -0.01 ± 0.07 | 0.10 | -0.07 to 0.26 | 0.24 |
| Interferon gamma (IFN-gamma) | -0.23 ± 0.19 | -0.09 ± 0.30 | -0.13 | -0.56 to 0.30 | 0.54 |
| Interleukin-1 alpha (IL-1 alpha) | 0.07 ± 0.07 | 0.15 ± 0.18 | -0.08 | -0.47 to 0.31 | 0.67 |
| Interleukin-10 (IL-10) | 0.05 ± 0.07 | 0.07 ± 0.09 | -0.02 | -0.17 to 0.13 | 0.77 |
| Interleukin-10 receptor subunit alpha (IL-10RA) | -0.06 ± 0.05 | -0.01 ± 0.04 | -0.05 | -0.13 to 0.31 | 0.22 |
| Interleukin-10 receptor subunit beta (IL-10RB) | 0.05 ± 0.04 | 0.04 ± 0.04 | 0.01 | -0.07 to 0.08 | 0.83 |
| Interleukin-12 subunit beta (IL-12B) | 0.03 ± 0.05 | 0.04 ± 0.05 | -0.01 | -0.12 to 0.11 | 0.93 |
| Interleukin-13 (IL-13) | -0.02 ± 0.07 | -0.01 ± 0.05 | -0.01 | -0.16 to 0.13 | 0.79 |
| Interleukin-15 subunit alpha (IL-15RA) | 0.06 ± 0.05 | 0.07 ± 0.04 | -0.01 | -0.08 to 0.08 | 0.94 |
| Interleukin-17A (IL-17A) | 0.07 ± 0.75 | 0.03 ± 0.76 | 0.04 | -0.14 to 0.23 | 0.64 |
| Interleukin-17C (IL-17C) | 0.11 ± 0.11 | 0.09 ± 0.10 | 0.03 | -0.23 to 0.28 | 0.83 |
| Interleukin-18 (IL-18) | 0.05 ± 0.05 | 0.07 ± 0.05 | -0.02 | -0.12 to 0.08 | 0.66 |
| Interleukin-18 receptor 1 (IL-18R1) | -0.02 ± 0.05 | -0.01 ± 0.05 | -0.01 | -0.10 to 0.08 | 0.85 |
| Interleukin-2 (IL-2) | 0.05 ± 0.06 | -0.01 ± 0.06 | 0.06 | -0.09 to 0.21 | 0.40 |
| Interleukin-20 (IL-20) | 0.03 ± 0.05 | -0.01 ± 0.05 | 0.04 | -0.05 to 0.13 | 0.36 |
| Interleukin-20 subunit alpha (IL-20RA) | -0.01 ± 0.06 | 0.08 ± 0.06 | -0.09 | -0.19 to 0.17 | 0.10 |
| Interleukin-22 subunit alpha-1 (IL-22RA1) | 0.28 ± 0.11 | 0.21 ± 0.09 | 0.07 | -0.15 to 0.28 | 0.54 |
| Interleukin-24 (IL-24) | -0.02 ± 0.11 | -0.17 ± 0.15 | 0.15 | -0.11 to 0.42 | 0.25 |
| Interleukin-2 receptor subunit beta (IL-2RB) | -0.04 ± 0.07 | -0.03 ± 0.08 | -0.02 | -0.17 to 0.13 | 0.83 |
| Interleukin-33 (IL-33) | -0.04 ± 0.07 | 0.05 ± 0.06 | -0.09 | -0.23 to 0.06 | 0.23 |
| Interleukin-4 (IL-4) | -0.02 ± 0.12 | -0.04 ± 0.08 | -0.02 | -0.18 to 0.12 | 0.88 |
| Interleukin-5 (IL-5) | -0.12 ± 0.11 | 0.07 ± 0.10 | -0.19 | -0.47 to 0.10 | 0.20 |
| Interleukin-6 (IL-6) | 0.09 ± 0.11 | 0.10 ± 0.12 | -0.01 | -0.21 to 0.20 | 0.95 |
| Interleukin-7 (IL-7) | 0.16 ± 0.07 | 0.08 ± 0.07 | 0.08 | -0.07 to 0.23 | 0.32 |
| Interleukin-8 (IL-8) | 0.10 ± 0.09 | 0.09 ± 0.09 | 0.01 | -0.21 to 0.22 | 0.94 |
| Latency-associated peptide transforming growth factor beta-1 (LAPTGF-beta-1) | 0.04 ± 0.07 | -0.01 ± 0.06 | 0.04 | -0.12 to 0.20 | 0.30 |
| Leukemia inhibitory factor receptor (LIF-R) | 0.05 ± 0.04 | 0.06 ± 0.03 | -0.01 | -0.09 to 0.07 | 0.81 |
| Leukemia inhibitory factor (LIF) | 0.03 ± 0.04 | -0.01 ± 0.05 | 0.04 | -0.06 to 0.14 | 0.47 |
| Macrophage colony-stimulating factor 1 (CSF-1) | -0.02 ± 0.08 | 0.03 ± 0.09 | -0.05 | -0.07 to 0.09 | 0.71 |
| Monocyte chemotactic protein 2 (MCP-2) | -0.04 ± 0.09 | -0.01 ± 0.11 | -0.03 | -0.24 to 0.19 | 0.80 |
| Monocyte chemotactic protein 3 (MCP-3) | 0.08 ± 0.06 | 0.15 ± 0.10 | -0.07 | -0.26 to 0.12 | 0.47 |
| Monocyte chemotactic protein 4 (MCP-4) | -0.01 ± 0.09 | -0.02 ± 0.10 | 0.01 | -0.23 to 0.26 | 0.89 |
| Matrix metalloproteinase-1 (MMP-1) | 0.03 ± 0.10 | -0.07 ± 0.10 | 0.10 | -0.13 to 0.34 | 0.37 |
| Matrix metalloproteinase-10 (MMP-10) | -0.04 ± 0.15 | -0.08 ± 0.14 | 0.04 | -0.26 to 0.34 | 0.79 |
| Neuturin (NRTN) | 0.01 ± 0.06 | 0.13 ± 0.07 | -0.12 | -0.28 to 0.03 | 0.12 |
| Neurotrophin-3 (NT-3) | 0.17 ± 0.07 | 0.12 ± 0.07 | 0.05 | -0.10 to 0.21 | 0.48 |
| Osteoprotegerin (OPG) | 0.08 ± 0.04 | 0.05 ± 0.05 | 0.03 | -0.07 to 0.12 | 0.58 |
| Oncostatin-M (OSM) | 0.21 ± 0.13 | 0.03 ± 0.16 | 0.18 | -0.15 to 0.50 | 0.27 |
| Programmed cell death 1 ligand 1 (PD-L1) | 0.01 ± 0.04 | 0.02 ± 0.05 | -0.01 | -0.10 to 0.09 | 0.89 |
| Stem cell factor (SCF) | 0.06 ± 0.04 | -0.04 ± 0.05 | 0.10 | -0.01 to 0.21 | 0.06 |
| SIR-like protein 2 (SIRT2) | 0.12 ± 0.17 | 0.16 ± 0.14 | -0.04 | -0.46 to 0.38 | 0.84 |
| Signaling lympohcytic activation molecule (SLAMF1) | 0.04 ± 0.06 | -0.05 ± 0.06 | -0.09 | -0.02 to 0.21 | 0.11 |
| Sulfotransferase 1A1 (ST1A1) | 0.12 ± 0.13 | 0.03 ± 0.12 | 0.09 | -0.17 to 0.35 | 0.48 |
| STAM-binding protein (STAMBP) | 0.23 ± 0.14 | 0.09 ± 0.11 | 0.14 | -0.16 to 0.44 | 0.33 |
| Transforming growth factor alpha (TGF-alpha) | 0.10 ± 0.09 | 0.01 ± 0.12 | 0.09 | -0.16 to 0.36 | 0.45 |
| Tumor necrosis factor (TNF) | 0.05 ± 0.11 | 0.06 ± 0.11 | -0.01 | -0.12 to 0.09 | 0.80 |
| TNF-beta (TNF-β) | -0.09 ± 0.05 | -0.02 ± 0.05 | -0.07 | -0.16 to 0.02 | 0.14 |
| Tumor necrosis factor receptor superfamily member 9 (TNFRSF9) | 0.05 ± 0.04 | 0.00 ± 0.04 | 0.05 | -0.05 to 0.15 | 0.30 |
| Tumor necrosis factor ligand superfamily member 14 (TNFSF14) | 0.06 ± 0.14 | -0.10 ± 0.15 | 0.16 | -0.18 to 0.51 | 0.33 |
| TNF-related apoptosis-inducing ligand (TRAIL) | -0.04 ± 0.34 | -0.02 ± 0.52 | -0.02 | -0.12 to 0.08 | 0.66 |
| TNF-related activation-induced cytokine (TRANCE) | -0.14 ± 0.10 | 0.02 ± 0.10 | -0.16 | -0.33 to 0.17 | 0.08 |
| Thymic stromal lymphopoietin (TSLP) | -0.01 ± 0.10 | -0.16 ± 0.13 | 0.16 | -0.11 to 0.42 | 0.23 |
| Tumor necrosis factor Ligand superfamily, member12 (TWEAK) | 0.00 ± 0.06 | 0.02 ± 0.05 | -0.02 | -0.16 to 0.12 | 0.75 |
| Urokinase-type plasminogen activator (uPA) | 0.03 ± 0.04 | 0.04 ± 0.04 | -0.01 | -0.01 to 0.09 | 0.94 |
| Vascular endothelial growth factor A (VEGF-A) | 0.09 ± 0.06 | 0.02 ± 0.06 | 0.07 | -0.08 to 0.22 | 0.34 |

^a^Biomarker units are normalized protein expression (NPX)

^b^P-value for Likelihood ratio test from mixed-effects model evaluating change scores for each product type (Plant compared with Animal), adjusting for order and phase.

**Supplementary Table 3** Results of liner mixed effects models comparing baseline values vs plant-based meat values (week 8) of all inflammatory biomarkers.^a^

| **Fixed Effects** | | | | | **Random Effects** | | | |  |
| --- | --- | --- | --- | --- | --- | --- | --- | --- | --- |
| **Variables** | **Parameters** | **Estimate** | **SE** | **T-value** | **P-Values^b^** | **Group** | **Parameters** | **Variance** | **SD** |
| Adenosine Deaminase (ADA) | Intercept | 4.21 | 0.07 | 61.59 | 0.00 | Participant ID | (Intercept) | 0.12 | 0.34 |
|  | Plant-based phase | 0.08 | 0.05 | 1.54 | 0.13 | Residual |  | 0.05 | 0.22 |
| Aretmin (ARTN) | Intercept | -0.20 | 0.07 | -3.02 | 0.00 | Participant ID | (Intercept) | 0.08 | 0.29 |
|  | Plant-based phase | -0.03 | 0.07 | -0.49 | 0.63 | Residual |  | 0.07 | 0.27 |
| Axin-1 (AXIN1) | Intercept | 0.37 | 0.12 | 2.98 | 0.00 | Participant ID | (Intercept) | 0.13 | 0.36 |
|  | Plant-based phase | 0.18 | 0.15 | 1.21 | 0.23 | Residual |  | 0.40 | 0.63 |
| Beta-nerve growth factor (Beta-NGF) | Intercept | -0.50 | 0.02 | -26.16 | 0.00 | Participant ID | (Intercept) | 0.00 | 0.04 |
|  | Plant-based phase | 0.01 | 0.03 | 0.41 | 0.69 | Residual |  | 0.01 | 0.11 |
| Caspase-8 (CASP-8) | Intercept | 0.49 | 0.07 | 7.08 | 0.00 | Participant ID | (Intercept) | 0.08 | 0.27 |
|  | Plant-based phase | 0.05 | 0.07 | 0.68 | 0.50 | Residual |  | 0.09 | 0.30 |
| Eotaxin (CCL11) | Intercept | 7.03 | 0.08 | 93.74 | 0.00 | Participant ID | (Intercept) | 0.12 | 0.35 |
|  | Plant-based phase | 0.03 | 0.07 | 0.46 | 0.65 | Residual |  | 0.07 | 0.27 |
| C-C motif chemokine 19 (CCL19) | Intercept | 8.41 | 0.13 | 65.16 | 0.00 | Participant ID | (Intercept) | 0.42 | 0.65 |
|  | Plant-based phase | 0.01 | 0.10 | 0.09 | 0.93 | Residual |  | 0.16 | 0.40 |
| C-C motif chemokine 20 (CCL20) | Intercept | 5.74 | 0.12 | 49.05 | 0.00 | Participant ID | (Intercept) | 0.17 | 0.41 |
|  | Plant-based phase | 0.05 | 0.13 | 0.38 | 0.71 | Residual |  | 0.31 | 0.56 |
| C-C motif chemokine 23 (CCL23) | Intercept | 9.57 | 0.08 | 117.11 | 0.00 | Participant ID | (Intercept) | 0.14 | 0.38 |
|  | Plant-based phase | 0.01 | 0.07 | 0.10 | 0.92 | Residual |  | 0.09 | 0.30 |
| C-C motif chemokine 25 (CCL25) | Intercept | 4.99 | 0.10 | 51.09 | 0.00 | Participant ID | (Intercept) | 0.29 | 0.54 |
|  | Plant-based phase | 0.10 | 0.05 | 1.92 | 0.06 | Residual |  | 0.04 | 0.21 |
| C-C motif chemokine 28 (CCL28) | Intercept | 0.92 | 0.06 | 15.62 | 0.00 | Participant ID | (Intercept) | 0.07 | 0.26 |
|  | Plant-based phase | 0.06 | 0.06 | 1.06 | 0.30 | Residual |  | 0.06 | 0.23 |
| C-C motif chemokine 3 (CCL3)(mean (SD)) | Intercept | 5.19 | 0.09 | 59.05 | 0.00 | Participant ID | (Intercept) | 0.18 | 0.43 |
|  | Plant-based phase | -0.03 | 0.07 | -0.42 | 0.68 | Residual |  | 0.09 | 0.30 |
| C-C motif chemokine 4 (CCL4) | Intercept | 6.04 | 0.10 | 59.58 | 0.00 | Participant ID | (Intercept) | 0.26 | 0.51 |
|  | Plant-based phase | -0.05 | 0.08 | -0.61 | 0.55 | Residual |  | 0.10 | 0.32 |
| Natural killer cell receptor 2B4 (CD244) | Intercept | 4.69 | 0.05 | 92.09 | 0.00 | Participant ID | (Intercept) | 0.07 | 0.26 |
|  | Plant-based phase | 0.06 | 0.04 | 1.54 | 0.13 | Residual |  | 0.02 | 0.15 |
| CD40L receptor (CD40) | Intercept | 9.86 | 0.06 | 171.65 | 0.00 | Participant ID | (Intercept) | 0.07 | 0.27 |
|  | Plant-based phase | 0.04 | 0.05 | 0.78 | 0.44 | Residual |  | 0.04 | 0.20 |
| T-cell surface glycoprotein CD5 (CD5) | Intercept | 4.10 | 0.05 | 78.74 | 0.00 | Participant ID | (Intercept) | 0.07 | 0.26 |
|  | Plant-based phase | 0.02 | 0.04 | 0.50 | 0.62 | Residual |  | 0.03 | 0.16 |
| T-cell surface glycoprotein CD6 isoform (CD6) | Intercept | 4.65 | 0.08 | 61.95 | 0.00 | Participant ID | (Intercept) | 0.15 | 0.38 |
|  | Plant-based phase | 0.07 | 0.05 | 1.28 | 0.21 | Residual |  | 0.05 | 0.22 |
| T cell surface glycoprotein CD8 alpha chain (CD8A) | Intercept | 7.96 | 0.11 | 75.17 | 0.00 | Participant ID | (Intercept) | 0.34 | 0.59 |
|  | Plant-based phase | 0.02 | 0.05 | 0.35 | 0.73 | Residual |  | 0.05 | 0.22 |
| CUB domain-containing protein 1 (CDCP1) | Intercept | 1.03 | 0.09 | 11.66 | 0.00 | Participant ID | (Intercept) | 0.23 | 0.48 |
|  | Plant-based phase | 0.08 | 0.05 | 1.54 | 0.13 | Residual |  | 0.05 | 0.21 |
| Cystatin D (CST5) | Intercept | 5.34 | 0.09 | 62.28 | 0.00 | Participant ID | (Intercept) | 0.22 | 0.47 |
|  | Plant-based phase | 0.06 | 0.04 | 1.39 | 0.17 | Residual |  | 0.03 | 0.18 |
| Fractalkine (CX3CL1) | Intercept | 2.77 | 0.05 | 53.31 | 0.00 | Participant ID | (Intercept) | 0.04 | 0.21 |
|  | Plant-based phase | 0.05 | 0.05 | 0.84 | 0.40 | Residual |  | 0.05 | 0.22 |
| C-X-C motif chemokine 1 (CXCL1) | Intercept | 8.78 | 0.14 | 64.92 | 0.00 | Participant ID | (Intercept) | 0.48 | 0.69 |
|  | Plant-based phase | 0.03 | 0.10 | 0.31 | 0.76 | Residual |  | 0.16 | 0.40 |
| C-X-C motif chemokine 10 (CXCL10) | Intercept | 9.04 | 0.10 | 91.92 | 0.00 | Participant ID | (Intercept) | 0.15 | 0.39 |
|  | Plant-based phase | -0.07 | 0.10 | -0.66 | 0.51 | Residual |  | 0.19 | 0.43 |
| C-X-C motif chemokine 11 (CXCL11) | Intercept | 6.72 | 0.13 | 51.35 | 0.00 | Participant ID | (Intercept) | 0.34 | 0.59 |
|  | Plant-based phase | -0.15 | 0.12 | -1.26 | 0.22 | Residual |  | 0.26 | 0.51 |
| C-X-C motif chemokine 5 (CXCL5) | Intercept | 11.51 | 0.13 | 89.41 | 0.00 | Participant ID | (Intercept) | 0.48 | 0.70 |
|  | Plant-based phase | -0.03 | 0.07 | -0.40 | 0.69 | Residual |  | 0.10 | 0.31 |
| C-X-C motif chemokine 6 (CXCL6) | Intercept | 7.95 | 0.13 | 61.31 | 0.00 | Participant ID | (Intercept) | 0.48 | 0.69 |
|  | Plant-based phase | -0.07 | 0.08 | -0.87 | 0.39 | Residual |  | 0.11 | 0.33 |
| C-X-C motif chemokine 9 (CXCL9) | Intercept | 5.59 | 0.11 | 52.38 | 0.00 | Participant ID | (Intercept) | 0.27 | 0.52 |
|  | Plant-based phase | -0.01 | 0.09 | -0.11 | 0.92 | Residual |  | 0.13 | 0.36 |
| Delta and Notch-like epidermal growth factor-related receptor (DNER) | Intercept | 7.57 | 0.04 | 173.10 | 0.00 | Participant ID | (Intercept) | 0.04 | 0.20 |
|  | Plant-based phase | 0.04 | 0.04 | 1.12 | 0.27 | Residual |  | 0.03 | 0.16 |
| Protein S100-A12 (EN-RAGE) | Intercept | 3.62 | 0.16 | 22.65 | 0.00 | Participant ID | (Intercept) | 0.51 | 0.71 |
|  | Plant-based phase | 0.12 | 0.15 | 0.82 | 0.42 | Residual |  | 0.38 | 0.62 |
| Fibroblast growth factor 19 (FGF-19) | Intercept | 7.32 | 0.16 | 46.91 | 0.00 | Participant ID | (Intercept) | 0.51 | 0.71 |
|  | Plant-based phase | -0.09 | 0.14 | -0.64 | 0.53 | Residual |  | 0.35 | 0.59 |
| Fibroblast growth factor 21 (FGF-21) | Intercept | 3.87 | 0.25 | 15.69 | 0.00 | Participant ID | (Intercept) | 1.29 | 1.14 |
|  | Plant-based phase | -0.03 | 0.22 | -0.13 | 0.90 | Residual |  | 0.84 | 0.92 |
| Fibroblast growth factor 23 (FGF-23) | Intercept | -0.11 | 0.05 | -2.36 | 0.02 | Participant ID | (Intercept) | 0.03 | 0.17 |
|  | Plant-based phase | -0.01 | 0.05 | -0.29 | 0.77 | Residual |  | 0.04 | 0.20 |
| Fibroblast growth factor 5 (FGF-5) | Intercept | -0.10 | 0.05 | -1.93 | 0.06 | Participant ID | (Intercept) | 0.06 | 0.25 |
|  | Plant-based phase | 0.06 | 0.04 | 1.55 | 0.13 | Residual |  | 0.03 | 0.16 |
| Fms-related tyrosine-kinase 3 ligand (FLT3L) | Intercept | 7.78 | 0.08 | 97.63 | 0.00 | Participant ID | (Intercept) | 0.19 | 0.43 |
|  | Plant-based phase | 0.09 | 0.05 | 1.95 | **0.06** | Residual |  | 0.04 | 0.19 |
| Eukaryotic translation initiation factor 4E-binding protein 1 (4E-BP1) | Intercept | 6.43 | 0.19 | 34.79 | 0.00 | Participant ID | (Intercept) | 0.34 | 0.58 |
|  | Plant-based phase | 0.29 | 0.22 | 1.30 | 0.20 | Residual |  | 0.86 | 0.93 |
| Glial cell line-dervied neurotrophic factor (GDNF) | Intercept | 1.02 | 0.08 | 13.32 | 0.00 | Participant ID | (Intercept) | 0.04 | 0.19 |
|  | Plant-based phase | -0.11 | 0.10 | -1.13 | 0.27 | Residual |  | 0.17 | 0.41 |
| Hepatocyte growth factor (HGF) | Intercept | 7.67 | 0.08 | 96.11 | 0.00 | Participant ID | (Intercept) | 0.16 | 0.40 |
|  | Plant-based phase | 0.09 | 0.06 | 1.42 | 0.16 | Residual |  | 0.06 | 0.25 |
| Interferon gamma (IFN-gamma) | Intercept | 6.46 | 0.19 | 34.35 | 0.00 | Participant ID | (Intercept) | 0.61 | 0.78 |
|  | Plant-based phase | -0.22 | 0.19 | -1.17 | 0.25 | Residual |  | 0.63 | 0.79 |
| Interleukin-1 alpha (IL-1 alpha) | Intercept | -0.63 | 0.07 | -9.46 | 0.00 | Participant ID | (Intercept) | 0.08 | 0.28 |
|  | Plant-based phase | 0.07 | 0.07 | 1.05 | 0.30 | Residual |  | 0.07 | 0.27 |
| Interleukin-10 (IL-10) | Intercept | 2.18 | 0.06 | 35.63 | 0.00 | Participant ID | (Intercept) | 0.05 | 0.23 |
|  | Plant-based phase | 0.05 | 0.07 | 0.70 | 0.49 | Residual |  | 0.08 | 0.28 |
| Interleukin-10 receptor subunit alpha (IL-10RA) | Intercept | -0.21 | 0.13 | -1.60 | 0.12 | Participant ID | (Intercept) | 0.58 | 0.76 |
|  | Plant-based phase | -0.06 | 0.05 | -1.29 | 0.21 | Residual |  | 0.04 | 0.19 |
| Interleukin-10 receptor subunit beta (IL-10RB) | Intercept | 5.10 | 0.05 | 96.48 | 0.00 | Participant ID | (Intercept) | 0.07 | 0.27 |
|  | Plant-based phase | 0.05 | 0.04 | 1.34 | 0.19 | Residual |  | 0.02 | 0.15 |
| Interleukin-12 subunit beta (IL-12B) | Intercept | 4.77 | 0.09 | 51.98 | 0.00 | Participant ID | (Intercept) | 0.25 | 0.50 |
|  | Plant-based phase | 0.03 | 0.05 | 0.56 | 0.58 | Residual |  | 0.04 | 0.21 |
| Interleukin-13 (IL-13) | Intercept | -0.20 | 0.11 | -1.79 | 0.08 | Participant ID | (Intercept) | 0.35 | 0.59 |
|  | Plant-based phase | -0.02 | 0.07 | -0.35 | 0.73 | Residual |  | 0.09 | 0.30 |
| Interleukin-15 subunit alpha (IL-15RA) | Intercept | -0.24 | 0.05 | -4.73 | 0.00 | Participant ID | (Intercept) | 0.05 | 0.22 |
|  | Plant-based phase | 0.06 | 0.05 | 1.32 | 0.20 | Residual |  | 0.04 | 0.20 |
| Interleukin-17A (IL-17A) | Intercept | 0.26 | 0.13 | 1.96 | 0.06 | Participant ID | (Intercept) | 0.50 | 0.71 |
|  | Plant-based phase | 0.07 | 0.08 | 0.94 | 0.36 | Residual |  | 0.10 | 0.31 |
| Interleukin-17C (IL-17C) | Intercept | 1.90 | 0.14 | 13.90 | 0.00 | Participant ID | (Intercept) | 0.44 | 0.66 |
|  | Plant-based phase | 0.11 | 0.11 | 1.02 | 0.32 | Residual |  | 0.21 | 0.46 |
| Interleukin-18 (IL-18) | Intercept | 7.85 | 0.10 | 82.64 | 0.00 | Participant ID | (Intercept) | 0.27 | 0.52 |
|  | Plant-based phase | 0.05 | 0.05 | 1.07 | 0.29 | Residual |  | 0.04 | 0.21 |
| Interleukin-18 receptor 1 (IL-18R1) | Intercept | 7.22 | 0.07 | 109.63 | 0.00 | Participant ID | (Intercept) | 0.11 | 0.33 |
|  | Plant-based phase | -0.02 | 0.05 | -0.32 | 0.75 | Residual |  | 0.04 | 0.21 |
| Interleukin-2 (IL-2) | Intercept | -0.48 | 0.05 | -9.80 | 0.00 | Participant ID | (Intercept) | 0.01 | 0.10 |
|  | Plant-based phase | 0.06 | 0.06 | 0.87 | 0.39 | Residual |  | 0.07 | 0.27 |
| Interleukin-20 (IL-20) | Intercept | -0.94 | 0.06 | -17.20 | 0.00 | Participant ID | (Intercept) | 0.06 | 0.25 |
|  | Plant-based phase | 0.03 | 0.05 | 0.61 | 0.55 | Residual |  | 0.04 | 0.21 |
| Interleukin-20 subunit alpha (IL-20RA) | Intercept | -0.48 | 0.06 | -8.35 | 0.00 | Participant ID | (Intercept) | 0.04 | 0.21 |
|  | Plant-based phase | -0.01 | 0.06 | -0.14 | 0.89 | Residual |  | 0.07 | 0.26 |
| Interleukin-22 subunit alpha-1 (IL-22RA1) | Intercept | 0.47 | 0.10 | 4.84 | 0.00 | Participant ID | (Intercept) | 0.13 | 0.37 |
|  | Plant-based phase | 0.28 | 0.11 | 2.61 | **0.01** | Residual |  | 0.20 | 0.45 |
| Interleukin-24 (IL-24) | Intercept | -0.19 | 0.15 | -1.22 | 0.23 | Participant ID | (Intercept) | 0.59 | 0.77 |
|  | Plant-based phase | -0.02 | 0.11 | -0.17 | 0.87 | Residual |  | 0.23 | 0.48 |
| Interleukin-2 receptor subunit beta (IL-2RB) | Intercept | -0.16 | 0.07 | -2.41 | 0.02 | Participant ID | (Intercept) | 0.07 | 0.27 |
|  | Plant-based phase | -0.04 | 0.07 | -0.65 | 0.52 | Residual |  | 0.08 | 0.28 |
| Interleukin-33 (IL-33) | Intercept | -0.92 | 0.06 | -16.32 | 0.00 | Participant ID | (Intercept) | 0.02 | 0.14 |
|  | Plant-based phase | -0.04 | 0.07 | -0.53 | 0.60 | Residual |  | 0.09 | 0.30 |
| Interleukin-4 (IL-4) | Intercept | -1.18 | 0.08 | -14.14 | 0.00 | Participant ID | (Intercept) | 0.01 | 0.09 |
|  | Plant-based phase | -0.02 | 0.12 | -0.19 | 0.85 | Residual |  | 0.23 | 0.48 |
| Interleukin-5 (IL-5) | Intercept | -0.54 | 0.11 | -4.73 | 0.00 | Participant ID | (Intercept) | 0.25 | 0.50 |
|  | Plant-based phase | -0.12 | 0.11 | -1.10 | 0.28 | Residual |  | 0.20 | 0.45 |
| Interleukin-6 (IL-6) | Intercept | 0.97 | 0.10 | 9.81 | 0.00 | Participant ID | (Intercept) | 0.12 | 0.34 |
|  | Plant-based phase | 0.09 | 0.11 | 0.79 | 0.43 | Residual |  | 0.22 | 0.47 |
| Interleukin-7 (IL-7) | Intercept | 2.48 | 0.08 | 32.10 | 0.00 | Participant ID | (Intercept) | 0.13 | 0.36 |
|  | Plant-based phase | 0.16 | 0.07 | 2.31 | **0.03** | Residual |  | 0.08 | 0.28 |
| Interleukin-8 (IL-8) | Intercept | 5.22 | 0.10 | 54.77 | 0.00 | Participant ID | (Intercept) | 0.18 | 0.43 |
|  | Plant-based phase | 0.10 | 0.09 | 1.14 | 0.26 | Residual |  | 0.14 | 0.37 |
| Latency-associated peptide transforming growth factor beta-1 (LAPTGF-beta-1) | Intercept | 4.92 | 0.07 | 73.05 | 0.00 | Participant ID | (Intercept) | 0.08 | 0.28 |
|  | Plant-based phase | 0.04 | 0.07 | 0.57 | 0.57 | Residual |  | 0.08 | 0.28 |
| Leukemia inhibitory factor receptor (LIF-R) | Intercept | -1.34 | 0.07 | -19.48 | 0.00 | Participant ID | (Intercept) | 0.13 | 0.36 |
|  | Plant-based phase | 0.03 | 0.04 | 0.67 | 0.51 | Residual |  | 0.03 | 0.18 |
| Leukemia inhibitory factor (LIF) | Intercept | 2.78 | 0.04 | 73.22 | 0.00 | Participant ID | (Intercept) | 0.02 | 0.16 |
|  | Plant-based phase | 0.05 | 0.04 | 1.40 | 0.17 | Residual |  | 0.03 | 0.16 |
| Macrophage colony-stimulating factor 1 (CSF-1) | Intercept | 11.16 | 0.10 | 115.50 | 0.00 | Participant ID | (Intercept) | 0.23 | 0.48 |
|  | Plant-based phase | -0.02 | 0.08 | -0.29 | 0.77 | Residual |  | 0.10 | 0.31 |
| Monocyte chemotactic protein 2 (MCP-2) | Intercept | 8.47 | 0.13 | 63.46 | 0.00 | Participant ID | (Intercept) | 0.48 | 0.69 |
|  | Plant-based phase | -0.04 | 0.09 | -0.43 | 0.67 | Residual |  | 0.15 | 0.38 |
| Monocyte chemotactic protein 3 (MCP-3) | Intercept | 0.62 | 0.09 | 6.77 | 0.00 | Participant ID | (Intercept) | 0.14 | 0.38 |
|  | Plant-based phase | 0.08 | 0.09 | 0.88 | 0.38 | Residual |  | 0.16 | 0.39 |
| Monocyte chemotactic protein 4 (MCP-4) | Intercept | 14.17 | 0.13 | 105.39 | 0.00 | Participant ID | (Intercept) | 0.49 | 0.70 |
|  | Plant-based phase | -0.01 | 0.09 | -0.06 | 0.95 | Residual |  | 0.14 | 0.37 |
| Matrix metalloproteinase-1 (MMP-1) | Intercept | 13.91 | 0.15 | 94.72 | 0.00 | Participant ID | (Intercept) | 0.59 | 0.77 |
|  | Plant-based phase | 0.03 | 0.10 | 0.33 | 0.75 | Residual |  | 0.17 | 0.41 |
| Matrix metalloproteinase-10 (MMP-10) | Intercept | 8.49 | 0.13 | 68.11 | 0.00 | Participant ID | (Intercept) | 0.16 | 0.40 |
|  | Plant-based phase | -0.04 | 0.15 | -0.25 | 0.80 | Residual |  | 0.39 | 0.62 |
| Neuturin (NRTN) | Intercept | -0.73 | 0.10 | -7.46 | 0.00 | Participant ID | (Intercept) | 0.27 | 0.52 |
|  | Plant-based phase | 0.01 | 0.06 | 0.18 | 0.86 | Residual |  | 0.06 | 0.25 |
| Neurotrophin-3 (NT-3) | Intercept | 0.30 | 0.08 | 3.90 | 0.00 | Participant ID | (Intercept) | 0.13 | 0.36 |
|  | Plant-based phase | 0.17 | 0.07 | 2.62 | **0.01** | Residual |  | 0.08 | 0.28 |
| Osteoprotegerin (OPG) | Intercept | 9.37 | 0.07 | 143.28 | 0.00 | Participant ID | (Intercept) | 0.12 | 0.35 |
|  | Plant-based phase | 0.07 | 0.04 | 1.86 | 0.07 | Residual |  | 0.03 | 0.17 |
| Oncostatin-M (OSM) | Intercept | 4.23 | 0.17 | 24.63 | 0.00 | Participant ID | (Intercept) | 0.73 | 0.85 |
|  | Plant-based phase | 0.21 | 0.13 | 1.56 | 0.13 | Residual |  | 0.30 | 0.55 |
| Programmed cell death 1 ligand 1 (PD-L1) | Intercept | 4.24 | 0.06 | 65.87 | 0.00 | Participant ID | (Intercept) | 0.11 | 0.34 |
|  | Plant-based phase | 0.01 | 0.04 | 0.29 | 0.78 | Residual |  | 0.03 | 0.18 |
| Stem cell factor (SCF) | Intercept | 8.58 | 0.06 | 152.02 | 0.00 | Participant ID | (Intercept) | 0.08 | 0.28 |
|  | Plant-based phase | 0.06 | 0.05 | 1.31 | 0.20 | Residual |  | 0.03 | 0.19 |
| SIR-like protein 2 (SIRT2) | Intercept | 1.20 | 0.16 | 7.67 | 0.00 | Participant ID | (Intercept) | 0.38 | 0.61 |
|  | Plant-based phase | 0.12 | 0.17 | 0.74 | 0.46 | Residual |  | 0.48 | 0.69 |
| Signaling lympohcytic activation molecule (SLAMF1) | Intercept | 1.08 | 0.06 | 17.04 | 0.00 | Participant ID | (Intercept) | 0.08 | 0.29 |
|  | Plant-based phase | 0.04 | 0.06 | 0.70 | 0.49 | Residual |  | 0.06 | 0.24 |
| Sulfotransferase 1A1 (ST1A1) | Intercept | 0.37 | 0.14 | 2.74 | 0.01 | Participant ID | (Intercept) | 0.32 | 0.57 |
|  | Plant-based phase | 0.12 | 0.13 | 0.87 | 0.39 | Residual |  | 0.31 | 0.56 |
| STAM-binding protein (STAMBP) | Intercept | 2.23 | 0.11 | 20.13 | 0.00 | Participant ID | (Intercept) | 0.11 | 0.33 |
|  | Plant-based phase | 0.23 | 0.14 | 1.69 | 0.10 | Residual |  | 0.32 | 0.57 |
| Transforming growth factor alpha (TGF-alpha) | Intercept | 2.30 | 0.14 | 17.06 | 0.00 | Participant ID | (Intercept) | 0.48 | 0.69 |
|  | Plant-based phase | 0.10 | 0.09 | 1.07 | 0.29 | Residual |  | 0.16 | 0.39 |
| Tumor necrosis factor (TNF) | Intercept | 1.28 | 0.10 | 13.04 | 0.00 | Participant ID | (Intercept) | 0.14 | 0.37 |
|  | Plant-based phase | 0.05 | 0.11 | 0.45 | 0.66 | Residual |  | 0.20 | 0.44 |
| TNF-beta (TNF-β) | Intercept | 3.65 | 0.06 | 64.36 | 0.00 | Participant ID | (Intercept) | 0.07 | 0.27 |
|  | Plant-based phase | -0.09 | 0.05 | -1.84 | 0.08 | Residual |  | 0.04 | 0.20 |
| Tumor necrosis factor receptor superfamily member 9 (TNFRSF9) | Intercept | 5.65 | 0.08 | 72.53 | 0.00 | Participant ID | (Intercept) | 0.19 | 0.43 |
|  | Plant-based phase | 0.05 | 0.04 | 1.25 | 0.22 | Residual |  | 0.02 | 0.15 |
| Tumor necrosis factor ligand superfamily member 14 (TNFSF14) | Intercept | 3.56 | 0.14 | 25.12 | 0.00 | Participant ID | (Intercept) | 0.36 | 0.60 |
|  | Plant-based phase | 0.06 | 0.14 | 0.43 | 0.67 | Residual |  | 0.34 | 0.58 |
| TNF-related apoptosis-inducing ligand (TRAIL) | Intercept | 7.08 | 0.05 | 158.13 | 0.00 | Participant ID | (Intercept) | 0.05 | 0.22 |
|  | Plant-based phase | -0.04 | 0.03 | -1.07 | 0.29 | Residual |  | 0.02 | 0.14 |
| TNF-related activation-induced cytokine (TRANCE) | Intercept | 3.64 | 0.12 | 31.60 | 0.00 | Participant ID | (Intercept) | 0.29 | 0.54 |
|  | Plant-based phase | -0.14 | 0.10 | -1.39 | 0.18 | Residual |  | 0.17 | 0.41 |
| Thymic stromal lymphopoietin (TSLP) | Intercept | 0.18 | 0.10 | 1.70 | 0.10 | Participant ID | (Intercept) | 0.20 | 0.44 |
|  | Plant-based phase | -0.01 | 0.10 | -0.07 | 0.95 | Residual |  | 0.19 | 0.43 |
| Tumor necrosis factor Ligand superfamily, member12 (TWEAK) | Intercept | 8.23 | 0.05 | 151.85 | 0.00 | Participant ID | (Intercept) | 0.04 | 0.21 |
|  | Plant-based phase | -0.01 | 0.06 | -0.08 | 0.94 | Residual |  | 0.06 | 0.24 |
| Urokinase-type plasminogen activator (uPA) | Intercept | 8.94 | 0.05 | 190.92 | 0.00 | Participant ID | (Intercept) | 0.05 | 0.22 |
|  | Plant-based phase | 0.03 | 0.04 | 0.76 | 0.45 | Residual |  | 0.03 | 0.17 |
| Vascular endothelial growth factor A (VEGF-A) | Intercept | 10.18 | 0.12 | 86.71 | 0.00 | Participant ID | (Intercept) | 0.42 | 0.65 |
|  | Plant-based phase | 0.09 | 0.06 | 1.44 | 0.16 | Residual |  | 0.06 | 0.25 |

^a^Biomarker units are normalized protein expression (NPX)

^b^P-value for Likelihood ratio test from mixed-effects model evaluating change scores for each product type (Plant compared with Animal), adjusting for order and phase. Bolded p-values indicate significance at < 0.05.

**Supplementary Table 4.** Results of liner mixed effects models comparing baseline values vs animal meat values (week 8) of all inflammatory biomarkers.^a^

|  | **Fixed Effects** | | | | | **Random Effects** | | | |
| --- | --- | --- | --- | --- | --- | --- | --- | --- | --- |
| **Variables** | **Parameters** | **Estimate** | **SE** | **T-value** | **P-Values^b^** | **Group** | **Parameters** | **Variance** | **SD** |
| Adenosine Deaminase (ADA) | Intercept | 4.21 | 0.07 | 57.32 | 0.00 | Participant ID | (Intercept) | 0.15 | 0.39 |
|  | Animal phase | 0.06 | 0.05 | 1.25 | 0.22 | Residual |  | 0.04 | 0.19 |
| Aretmin (ARTN) | Intercept | -0.20 | 0.06 | -3.20 | 0.00 | Participant ID | (Intercept) | 0.07 | 0.27 |
|  | Animal phase | 0.01 | 0.06 | 0.23 | 0.82 | Residual |  | 0.07 | 0.26 |
| Axin-1 (AXIN1) | Intercept | 0.37 | 0.10 | 3.67 | 0.00 | Participant ID | (Intercept) | 0.04 | 0.20 |
|  | Animal phase | 0.00 | 0.13 | 0.01 | 0.99 | Residual |  | 0.31 | 0.56 |
| Beta-nerve growth factor (Beta-NGF) | Intercept | -0.50 | 0.02 | -27.35 | 0.00 | Participant ID | (Intercept) | 0.00 | 0.06 |
|  | Animal phase | -0.03 | 0.02 | -1.57 | 0.13 | Residual |  | 0.01 | 0.09 |
| Caspase-8 (CASP-8) | Intercept | 0.49 | 0.06 | 7.67 | 0.00 | Participant ID | (Intercept) | 0.09 | 0.30 |
|  | Animal phase | 0.06 | 0.05 | 1.01 | 0.32 | Residual |  | 0.05 | 0.23 |
| Eotaxin (CCL11) | Intercept | 7.03 | 0.08 | 93.82 | 0.00 | Participant ID | (Intercept) | 0.13 | 0.36 |
|  | Animal phase | 0.02 | 0.06 | 0.33 | 0.74 | Residual |  | 0.06 | 0.25 |
| C-C motif chemokine 19 (CCL19) | Intercept | 8.41 | 0.12 | 72.16 | 0.00 | Participant ID | (Intercept) | 0.24 | 0.49 |
|  | Animal phase | -0.15 | 0.12 | -1.26 | 0.22 | Residual |  | 0.23 | 0.48 |
| C-C motif chemokine 20 (CCL20) | Intercept | 5.74 | 0.13 | 43.82 | 0.00 | Participant ID | (Intercept) | 0.23 | 0.48 |
|  | Animal phase | 0.15 | 0.15 | 1.01 | 0.32 | Residual |  | 0.37 | 0.61 |
| C-C motif chemokine 23 (CCL23) | Intercept | 9.57 | 0.08 | 125.84 | 0.00 | Participant ID | (Intercept) | 0.12 | 0.34 |
|  | Animal phase | 0.05 | 0.07 | 0.75 | 0.46 | Residual |  | 0.09 | 0.29 |
| C-C motif chemokine 25 (CCL25) | Intercept | 4.99 | 0.10 | 49.50 | 0.00 | Participant ID | (Intercept) | 0.32 | 0.57 |
|  | Animal phase | 0.03 | 0.04 | 0.73 | 0.47 | Residual |  | 0.03 | 0.18 |
| C-C motif chemokine 28 (CCL28) | Intercept | 0.92 | 0.06 | 16.74 | 0.00 | Participant ID | (Intercept) | 0.03 | 0.16 |
|  | Animal phase | 0.01 | 0.07 | 0.14 | 0.89 | Residual |  | 0.08 | 0.28 |
| C-C motif chemokine 3 (CCL3) (mean (SD)) | Intercept | 5.19 | 0.09 | 55.11 | 0.00 | Participant ID | (Intercept) | 0.17 | 0.41 |
|  | Animal phase | -0.01 | 0.09 | -0.12 | 0.91 | Residual |  | 0.14 | 0.37 |
| C-C motif chemokine 4 (CCL4) | Intercept | 6.04 | 0.11 | 55.75 | 0.00 | Participant ID | (Intercept) | 0.22 | 0.47 |
|  | Animal phase | -0.04 | 0.11 | -0.37 | 0.72 | Residual |  | 0.19 | 0.44 |
| Natural killer cell receptor 2B4 (CD244) | Intercept | 4.69 | 0.06 | 85.04 | 0.00 | Participant ID | (Intercept) | 0.08 | 0.29 |
|  | Animal phase | 0.02 | 0.04 | 0.60 | 0.55 | Residual |  | 0.02 | 0.15 |
| CD40L receptor (CD40) | Intercept | 9.86 | 0.06 | 179.63 | 0.00 | Participant ID | (Intercept) | 0.07 | 0.26 |
|  | Animal phase | 0.03 | 0.05 | 0.66 | 0.51 | Residual |  | 0.04 | 0.20 |
| T-cell surface glycoprotein CD5 (CD5) | Intercept | 4.10 | 0.05 | 77.41 | 0.00 | Participant ID | (Intercept) | 0.08 | 0.28 |
|  | Animal phase | 0.03 | 0.04 | 0.75 | 0.46 | Residual |  | 0.02 | 0.15 |
| T-cell surface glycoprotein CD6 isoform (CD6) | Intercept | 4.65 | 0.08 | 57.76 | 0.00 | Participant ID | (Intercept) | 0.19 | 0.43 |
|  | Animal phase | 0.03 | 0.05 | 0.66 | 0.51 | Residual |  | 0.04 | 0.20 |
| T cell surface glycoprotein CD8 alpha chain (CD8A) | Intercept | 7.96 | 0.11 | 75.51 | 0.00 | Participant ID | (Intercept) | 0.35 | 0.59 |
|  | Animal phase | -0.01 | 0.05 | -0.11 | 0.91 | Residual |  | 0.04 | 0.20 |
| CUB domain-containing protein 1 (CDCP1) | Intercept | 1.03 | 0.09 | 11.36 | 0.00 | Participant ID | (Intercept) | 0.24 | 0.49 |
|  | Animal phase | 0.05 | 0.05 | 0.95 | 0.35 | Residual |  | 0.04 | 0.20 |
| Cystatin D (CST5) | Intercept | 5.34 | 0.09 | 59.76 | 0.00 | Participant ID | (Intercept) | 0.24 | 0.49 |
|  | Animal phase | 0.05 | 0.05 | 1.08 | 0.29 | Residual |  | 0.04 | 0.21 |
| Fractalkine (CX3CL1) | Intercept | 2.77 | 0.05 | 52.54 | 0.00 | Participant ID | (Intercept) | 0.05 | 0.21 |
|  | Animal phase | 0.07 | 0.05 | 1.33 | 0.19 | Residual |  | 0.05 | 0.23 |
| C-X-C motif chemokine 1 (CXCL1) | Intercept | 8.78 | 0.13 | 65.37 | 0.00 | Participant ID | (Intercept) | 0.42 | 0.65 |
|  | Animal phase | -0.08 | 0.11 | -0.73 | 0.47 | Residual |  | 0.21 | 0.46 |
| C-X-C motif chemokine 10 (CXCL10) | Intercept | 9.04 | 0.12 | 78.13 | 0.00 | Participant ID | (Intercept) | 0.06 | 0.25 |
|  | Animal phase | 0.04 | 0.15 | 0.23 | 0.82 | Residual |  | 0.40 | 0.64 |
| C-X-C motif chemokine 11 (CXCL11) | Intercept | 6.72 | 0.14 | 48.56 | 0.00 | Participant ID | (Intercept) | 0.28 | 0.53 |
|  | Animal phase | -0.11 | 0.15 | -0.76 | 0.45 | Residual |  | 0.39 | 0.63 |
| C-X-C motif chemokine 5 (CXCL5) | Intercept | 11.51 | 0.13 | 89.06 | 0.00 | Participant ID | (Intercept) | 0.45 | 0.67 |
|  | Animal phase | 0.07 | 0.09 | 0.77 | 0.45 | Residual |  | 0.13 | 0.36 |
| C-X-C motif chemokine 6 (CXCL6) | Intercept | 7.95 | 0.13 | 59.29 | 0.00 | Participant ID | (Intercept) | 0.47 | 0.68 |
|  | Animal phase | -0.04 | 0.10 | -0.38 | 0.71 | Residual |  | 0.16 | 0.40 |
| C-X-C motif chemokine 9 (CXCL9) | Intercept | 5.59 | 0.13 | 42.97 | 0.00 | Participant ID | (Intercept) | 0.26 | 0.51 |
|  | Animal phase | 0.04 | 0.14 | 0.32 | 0.75 | Residual |  | 0.34 | 0.58 |
| Delta and Notch-like epidermal growth factor-related receptor (DNER) | Intercept | 7.57 | 0.05 | 155.79 | 0.00 | Participant ID | (Intercept) | 0.06 | 0.25 |
|  | Animal phase | 0.04 | 0.04 | 1.19 | 0.24 | Residual |  | 0.02 | 0.15 |
| Protein S100-A12 (EN-RAGE) | Intercept | 3.62 | 0.15 | 23.45 | 0.00 | Participant ID | (Intercept) | 0.42 | 0.65 |
|  | Animal phase | 0.13 | 0.15 | 0.84 | 0.41 | Residual |  | 0.42 | 0.65 |
| Fibroblast growth factor 19 (FGF-19) | Intercept | 7.32 | 0.17 | 43.69 | 0.00 | Participant ID | (Intercept) | 0.44 | 0.67 |
|  | Animal phase | 0.08 | 0.18 | 0.45 | 0.66 | Residual |  | 0.54 | 0.73 |
| Fibroblast growth factor 21 (FGF-21) | Intercept | 3.87 | 0.25 | 15.40 | 0.00 | Participant ID | (Intercept) | 1.14 | 1.07 |
|  | Animal phase | -0.06 | 0.25 | -0.25 | 0.81 | Residual |  | 1.07 | 1.03 |
| Fibroblast growth factor 23 (FGF-23) | Intercept | -0.11 | 0.05 | -2.17 | 0.03 | Participant ID | (Intercept) | 0.03 | 0.16 |
|  | Animal phase | -0.05 | 0.06 | -0.82 | 0.42 | Residual |  | 0.06 | 0.24 |
| Fibroblast growth factor 5 (FGF-5) | Intercept | -0.10 | 0.05 | -1.96 | 0.06 | Participant ID | (Intercept) | 0.05 | 0.23 |
|  | Animal phase | 0.05 | 0.04 | 1.19 | 0.24 | Residual |  | 0.03 | 0.18 |
| Fms-related tyrosine-kinase 3 ligand (FLT3L) | Intercept | 7.78 | 0.08 | 103.13 | 0.00 | Participant ID | (Intercept) | 0.16 | 0.40 |
|  | Animal phase | 0.12 | 0.05 | 2.43 | **0.02** | Residual |  | 0.04 | 0.21 |
| Eukaryotic translation initiation factor 4E-binding protein 1 (4E-BP1) | Intercept | 6.43 | 0.17 | 38.35 | 0.00 | Participant ID | (Intercept) | 0.00 | 0.00 |
|  | Animal phase | 0.28 | 0.24 | 1.19 | 0.24 | Residual |  | 0.98 | 0.99 |
| Glial cell line-dervied neurotrophic factor (GDNF) | Intercept | 1.02 | 0.08 | 12.90 | 0.00 | Participant ID | (Intercept) | 0.04 | 0.19 |
|  | Animal phase | -0.09 | 0.10 | -0.90 | 0.38 | Residual |  | 0.18 | 0.42 |
| Hepatocyte growth factor (HGF) | Intercept | 7.67 | 0.08 | 100.37 | 0.00 | Participant ID | (Intercept) | 0.13 | 0.36 |
|  | Animal phase | -0.01 | 0.07 | -0.15 | 0.89 | Residual |  | 0.07 | 0.27 |
| Interferon gamma (IFN-gamma) | Intercept | 6.46 | 0.21 | 30.53 | 0.00 | Participant ID | (Intercept) | 0.00 | 0.00 |
|  | Animal phase | -0.09 | 0.30 | -0.30 | 0.76 | Residual |  | 1.57 | 1.25 |
| Interleukin-1 alpha (IL-1 alpha) | Intercept | -0.63 | 0.12 | -5.05 | 0.00 | Participant ID | (Intercept) | 0.00 | 0.00 |
|  | Animal phase | 0.15 | 0.18 | 0.87 | 0.39 | Residual |  | 0.54 | 0.73 |
| Interleukin-10 (IL-10) | Intercept | 2.18 | 0.07 | 32.18 | 0.00 | Participant ID | (Intercept) | 0.03 | 0.18 |
|  | Animal phase | 0.07 | 0.09 | 0.80 | 0.43 | Residual |  | 0.13 | 0.36 |
| Interleukin-10 receptor subunit alpha (IL-10RA) | Intercept | -0.21 | 0.13 | -1.60 | 0.12 | Participant ID | (Intercept) | 0.59 | 0.77 |
|  | Animal phase | -0.01 | 0.04 | -0.21 | 0.83 | Residual |  | 0.03 | 0.18 |
| Interleukin-10 receptor subunit beta (IL-10RB) | Intercept | 5.10 | 0.05 | 99.36 | 0.00 | Participant ID | (Intercept) | 0.07 | 0.26 |
|  | Animal phase | 0.04 | 0.04 | 1.08 | 0.29 | Residual |  | 0.02 | 0.16 |
| Interleukin-12 subunit beta (IL-12B) | Intercept | 4.77 | 0.09 | 54.00 | 0.00 | Participant ID | (Intercept) | 0.23 | 0.48 |
|  | Animal phase | 0.03 | 0.05 | 0.65 | 0.52 | Residual |  | 0.04 | 0.21 |
| Interleukin-13 (IL-13) | Intercept | -0.20 | 0.11 | -1.86 | 0.07 | Participant ID | (Intercept) | 0.36 | 0.60 |
|  | Animal phase | -0.01 | 0.05 | -0.11 | 0.91 | Residual |  | 0.04 | 0.21 |
| Interleukin-15 subunit alpha (IL-15RA) | Intercept | -0.24 | 0.05 | -4.93 | 0.00 | Participant ID | (Intercept) | 0.05 | 0.22 |
|  | Animal phase | 0.07 | 0.04 | 1.54 | 0.13 | Residual |  | 0.03 | 0.18 |
| Interleukin-17A (IL-17A) | Intercept | 0.26 | 0.13 | 1.90 | 0.06 | Participant ID | (Intercept) | 0.53 | 0.73 |
|  | Animal phase | 0.03 | 0.08 | 0.36 | 0.72 | Residual |  | 0.10 | 0.32 |
| Interleukin-17C (IL-17C) | Intercept | 1.90 | 0.14 | 13.64 | 0.00 | Participant ID | (Intercept) | 0.51 | 0.72 |
|  | Animal phase | 0.09 | 0.10 | 0.87 | 0.39 | Residual |  | 0.17 | 0.41 |
| Interleukin-18 (IL-18) | Intercept | 7.85 | 0.10 | 79.33 | 0.00 | Participant ID | (Intercept) | 0.31 | 0.55 |
|  | Animal phase | 0.07 | 0.05 | 1.63 | 0.11 | Residual |  | 0.04 | 0.19 |
| Interleukin-18 receptor 1 (IL-18R1) | Intercept | 7.22 | 0.07 | 109.60 | 0.00 | Participant ID | (Intercept) | 0.11 | 0.33 |
|  | Animal phase | -0.01 | 0.05 | -0.13 | 0.90 | Residual |  | 0.05 | 0.21 |
| Interleukin-2 (IL-2) | Intercept | -0.48 | 0.05 | -10.26 | 0.00 | Participant ID | (Intercept) | 0.01 | 0.11 |
|  | Animal phase | -0.01 | 0.06 | -0.12 | 0.91 | Residual |  | 0.06 | 0.25 |
| Interleukin-20 (IL-20) | Intercept | -0.94 | 0.05 | -18.36 | 0.00 | Participant ID | (Intercept) | 0.06 | 0.24 |
|  | Animal phase | -0.01 | 0.05 | -0.23 | 0.82 | Residual |  | 0.04 | 0.19 |
| Interleukin-20 subunit alpha (IL-20RA) | Intercept | -0.48 | 0.06 | -8.59 | 0.00 | Participant ID | (Intercept) | 0.05 | 0.21 |
|  | Animal phase | 0.08 | 0.06 | 1.33 | 0.19 | Residual |  | 0.06 | 0.25 |
| Interleukin-22 subunit alpha-1 (IL-22RA1) | Intercept | 0.47 | 0.10 | 4.62 | 0.00 | Participant ID | (Intercept) | 0.22 | 0.47 |
|  | Animal phase | 0.21 | 0.09 | 2.31 | **0.03** | Residual |  | 0.15 | 0.39 |
| Interleukin-24 (IL-24) | Intercept | -0.19 | 0.16 | -1.18 | 0.24 | Participant ID | (Intercept) | 0.50 | 0.71 |
|  | Animal phase | -0.17 | 0.15 | -1.18 | 0.25 | Residual |  | 0.37 | 0.61 |
| Interleukin-2 receptor subunit beta (IL-2RB) | Intercept | -0.16 | 0.07 | -2.43 | 0.02 | Participant ID | (Intercept) | 0.04 | 0.21 |
|  | Animal phase | -0.03 | 0.08 | -0.35 | 0.73 | Residual |  | 0.10 | 0.32 |
| Interleukin-33 (IL-33) | Intercept | -0.92 | 0.05 | -17.19 | 0.00 | Participant ID | (Intercept) | 0.04 | 0.19 |
|  | Animal phase | 0.05 | 0.06 | 0.79 | 0.44 | Residual |  | 0.06 | 0.25 |
| Interleukin-4 (IL-4) | Intercept | -1.18 | 0.08 | -15.45 | 0.00 | Participant ID | (Intercept) | 0.09 | 0.30 |
|  | Animal phase | -0.04 | 0.08 | -0.45 | 0.66 | Residual |  | 0.11 | 0.34 |
| Interleukin-5 (IL-5) | Intercept | -0.54 | 0.13 | -4.10 | 0.00 | Participant ID | (Intercept) | 0.44 | 0.67 |
|  | Animal phase | 0.07 | 0.10 | 0.68 | 0.50 | Residual |  | 0.16 | 0.40 |
| Interleukin-6 (IL-6) | Intercept | 0.97 | 0.11 | 8.90 | 0.00 | Participant ID | (Intercept) | 0.15 | 0.38 |
|  | Animal phase | 0.10 | 0.12 | 0.78 | 0.44 | Residual |  | 0.27 | 0.52 |
| Interleukin-7 (IL-7) | Intercept | 2.48 | 0.07 | 34.36 | 0.00 | Participant ID | (Intercept) | 0.10 | 0.31 |
|  | Animal phase | 0.08 | 0.07 | 1.11 | 0.27 | Residual |  | 0.08 | 0.29 |
| Interleukin-8 (IL-8) | Intercept | 5.22 | 0.10 | 54.73 | 0.00 | Participant ID | (Intercept) | 0.18 | 0.42 |
|  | Animal phase | 0.09 | 0.09 | 1.04 | 0.31 | Residual |  | 0.14 | 0.37 |
| Latency-associated peptide transforming growth factor beta-1 (LAPTGF-beta-1) | Intercept | 4.92 | 0.07 | 76.12 | 0.00 | Participant ID | (Intercept) | 0.07 | 0.27 |
|  | Animal phase | 0.00 | 0.07 | -0.04 | 0.97 | Residual |  | 0.07 | 0.27 |
| Leukemia inhibitory factor receptor (LIF-R) | Intercept | -1.34 | 0.07 | -20.21 | 0.00 | Participant ID | (Intercept) | 0.11 | 0.32 |
|  | Animal phase | -0.01 | 0.05 | -0.12 | 0.91 | Residual |  | 0.05 | 0.22 |
| Leukemia inhibitory factor (LIF) | Intercept | 2.78 | 0.04 | 80.32 | 0.00 | Participant ID | (Intercept) | 0.02 | 0.15 |
|  | Animal phase | 0.06 | 0.03 | 1.95 | 0.06 | Residual |  | 0.02 | 0.14 |
| Macrophage colony-stimulating factor 1 (CSF-1) | Intercept | 11.16 | 0.10 | 114.02 | 0.00 | Participant ID | (Intercept) | 0.21 | 0.45 |
|  | Animal phase | 0.03 | 0.09 | 0.40 | 0.70 | Residual |  | 0.13 | 0.36 |
| Monocyte chemotactic protein 2 (MCP-2) | Intercept | 8.47 | 0.13 | 65.85 | 0.00 | Participant ID | (Intercept) | 0.36 | 0.60 |
|  | Animal phase | -0.01 | 0.11 | -0.11 | 0.91 | Residual |  | 0.22 | 0.47 |
| Monocyte chemotactic protein 3 (MCP-3) | Intercept | 0.62 | 0.09 | 6.93 | 0.00 | Participant ID | (Intercept) | 0.10 | 0.31 |
|  | Animal phase | 0.15 | 0.10 | 1.48 | 0.15 | Residual |  | 0.18 | 0.43 |
| Monocyte chemotactic protein 4 (MCP-4) | Intercept | 14.17 | 0.13 | 108.67 | 0.00 | Participant ID | (Intercept) | 0.44 | 0.66 |
|  | Animal phase | -0.02 | 0.10 | -0.23 | 0.82 | Residual |  | 0.16 | 0.40 |
| Matrix metalloproteinase-1 (MMP-1) | Intercept | 13.91 | 0.14 | 99.52 | 0.00 | Participant ID | (Intercept) | 0.52 | 0.72 |
|  | Animal phase | -0.07 | 0.10 | -0.75 | 0.46 | Residual |  | 0.16 | 0.40 |
| Matrix metalloproteinase-10 (MMP-10) | Intercept | 8.49 | 0.13 | 65.82 | 0.00 | Participant ID | (Intercept) | 0.23 | 0.48 |
|  | Animal phase | -0.08 | 0.14 | -0.54 | 0.59 | Residual |  | 0.35 | 0.60 |
| Neuturin (NRTN) | Intercept | -0.73 | 0.09 | -8.03 | 0.00 | Participant ID | (Intercept) | 0.21 | 0.45 |
|  | Animal phase | 0.13 | 0.07 | 1.98 | 0.06 | Residual |  | 0.08 | 0.28 |
| Neurotrophin-3 (NT-3) | Intercept | 0.30 | 0.07 | 4.02 | 0.00 | Participant ID | (Intercept) | 0.11 | 0.33 |
|  | Animal phase | 0.12 | 0.07 | 1.66 | 0.11 | Residual |  | 0.09 | 0.30 |
| Osteoprotegerin (OPG) | Intercept | 9.37 | 0.07 | 135.96 | 0.00 | Participant ID | (Intercept) | 0.13 | 0.35 |
|  | Animal phase | 0.05 | 0.05 | 1.01 | 0.32 | Residual |  | 0.04 | 0.20 |
| Oncostatin-M (OSM) | Intercept | 4.23 | 0.17 | 24.99 | 0.00 | Participant ID | (Intercept) | 0.57 | 0.76 |
|  | Animal phase | 0.03 | 0.16 | 0.17 | 0.86 | Residual |  | 0.43 | 0.66 |
| Programmed cell death 1 ligand 1 (PD-L1) | Intercept | 4.24 | 0.07 | 63.84 | 0.00 | Participant ID | (Intercept) | 0.11 | 0.34 |
|  | Animal phase | 0.02 | 0.05 | 0.39 | 0.70 | Residual |  | 0.04 | 0.20 |
| Stem cell factor (SCF) | Intercept | 8.58 | 0.06 | 143.76 | 0.00 | Participant ID | (Intercept) | 0.08 | 0.28 |
|  | Animal phase | -0.04 | 0.05 | -0.84 | 0.41 | Residual |  | 0.05 | 0.22 |
| SIR-like protein 2 (SIRT2) | Intercept | 1.20 | 0.13 | 9.41 | 0.00 | Participant ID | (Intercept) | 0.24 | 0.49 |
|  | Animal phase | 0.17 | 0.14 | 1.20 | 0.24 | Residual |  | 0.33 | 0.57 |
| Signaling lympohcytic activation molecule (SLAMF1) | Intercept | 1.08 | 0.07 | 16.30 | 0.00 | Participant ID | (Intercept) | 0.08 | 0.29 |
|  | Animal phase | -0.05 | 0.06 | -0.82 | 0.42 | Residual |  | 0.07 | 0.26 |
| Sulfotransferase 1A1 (ST1A1) | Intercept | 0.37 | 0.13 | 2.83 | 0.01 | Participant ID | (Intercept) | 0.36 | 0.60 |
|  | Animal phase | 0.03 | 0.12 | 0.21 | 0.83 | Residual |  | 0.24 | 0.49 |
| STAM-binding protein (STAMBP) | Intercept | 2.23 | 0.09 | 25.67 | 0.00 | Participant ID | (Intercept) | 0.05 | 0.23 |
|  | Animal phase | 0.09 | 0.11 | 0.78 | 0.44 | Residual |  | 0.21 | 0.46 |
| Transforming growth factor alpha (TGF-alpha) | Intercept | 2.30 | 0.13 | 17.88 | 0.00 | Participant ID | (Intercept) | 0.32 | 0.56 |
|  | Animal phase | 0.00 | 0.12 | 0.03 | 0.98 | Residual |  | 0.26 | 0.51 |
| Tumor necrosis factor (TNF) | Intercept | 1.28 | 0.10 | 13.15 | 0.00 | Participant ID | (Intercept) | 0.12 | 0.35 |
|  | Animal phase | 0.06 | 0.11 | 0.56 | 0.58 | Residual |  | 0.21 | 0.46 |
| TNF-beta (TNF-β) | Intercept | 3.65 | 0.06 | 57.95 | 0.00 | Participant ID | (Intercept) | 0.10 | 0.32 |
|  | Animal phase | -0.02 | 0.05 | -0.45 | 0.65 | Residual |  | 0.04 | 0.19 |
| Tumor necrosis factor receptor superfamily member 9 (TNFRSF9) | Intercept | 5.65 | 0.08 | 69.02 | 0.00 | Participant ID | (Intercept) | 0.21 | 0.46 |
|  | Animal phase | 0.00 | 0.04 | -0.09 | 0.93 | Residual |  | 0.03 | 0.16 |
| Tumor necrosis factor ligand superfamily member 14 (TNFSF14) | Intercept | 3.56 | 0.14 | 25.99 | 0.00 | Participant ID | (Intercept) | 0.25 | 0.50 |
|  | Animal phase | -0.11 | 0.15 | -0.71 | 0.49 | Residual |  | 0.40 | 0.64 |
| TNF-related apoptosis-inducing ligand (TRAIL) | Intercept | 7.08 | 0.05 | 145.93 | 0.00 | Participant ID | (Intercept) | 0.03 | 0.18 |
|  | Animal phase | -0.02 | 0.05 | -0.29 | 0.78 | Residual |  | 0.05 | 0.22 |
| TNF-related activation-induced cytokine (TRANCE) | Intercept | 3.64 | 0.11 | 32.22 | 0.00 | Participant ID | (Intercept) | 0.28 | 0.53 |
|  | Animal phase | 0.02 | 0.10 | 0.20 | 0.84 | Residual |  | 0.17 | 0.41 |
| Thymic stromal lymphopoietin (TSLP) | Intercept | 0.18 | 0.11 | 1.55 | 0.13 | Participant ID | (Intercept) | 0.15 | 0.39 |
|  | Animal phase | -0.16 | 0.13 | -1.26 | 0.22 | Residual |  | 0.30 | 0.55 |
| Tumor necrosis factor Ligand superfamily, member12 (TWEAK) | Intercept | 8.23 | 0.06 | 149.33 | 0.00 | Participant ID | (Intercept) | 0.06 | 0.24 |
|  | Animal phase | 0.02 | 0.05 | 0.33 | 0.74 | Residual |  | 0.05 | 0.22 |
| Urokinase-type plasminogen activator (uPA) | Intercept | 8.94 | 0.05 | 168.03 | 0.00 | Participant ID | (Intercept) | 0.07 | 0.27 |
|  | Animal phase | 0.03 | 0.04 | 0.89 | 0.38 | Residual |  | 0.03 | 0.16 |
| Vascular endothelial growth factor A (VEGF-A) | Intercept | 10.18 | 0.12 | 88.18 | 0.00 | Participant ID | (Intercept) | 0.40 | 0.63 |
|  | Animal phase | 0.02 | 0.06 | 0.25 | 0.80 | Residual |  | 0.06 | 0.25 |

^a^Biomarker units are normalized protein expression (NPX)

^b^P-value for Likelihood ratio test from mixed-effects model evaluating change scores for each product type (Plant compared with Animal), adjusting for order and phase. Bolded p-values indicate significance at < 0.05
